# Emerging Nanomedicine Strategies for Sepsis: Immunomodulation and Beyond

**DOI:** 10.34133/research.1254

**Published:** 2026-04-22

**Authors:** Kerong Yang, Bingjie Liu, Yunpeng Bai, Shenglong Chen, Chunbo Chen, Jun-Bing Fan

**Affiliations:** ^1^ Cancer Research Institute, Experimental Education/Administration Center, School of Basic Medical Sciences, Southern Medical University, Guangzhou 510515, P. R. China.; ^2^ Department of Critical Care Medicine, Guangdong Provincial People’s Hospital, Guangdong Academy of Medical Sciences, Southern Medical University, Guangzhou, P.R. China.; ^3^ Department of Critical Care Medicine, Shenzhen People’s Hospital, The First Affiliated Hospital of Southern University of Science and Technology, The Second Clinical Medical College of Jinan University, Shenzhen, P.R. China.

## Abstract

Sepsis is defined as a life-threatening organ dysfunction caused by a dysregulated host immune response to infection, initiated by an excessive inflammatory cascade that frequently progresses to immunoparalysis characterized by immune cell dysfunction. In this review, we first provide an overview of the key elements and stages in the onset and progression of sepsis. Subsequently, we discuss the recent advances of biomaterial-based nanomedicine (including organic nanomaterials, inorganic nanomaterials, and natural bio-inspired nanomaterials) in the treatment of sepsis. The elimination of pathogens and the modulation of the immune response within the sepsis microenvironment through the design of diverse biomaterial systems were specifically summarized. Finally, we discuss the challenges and opportunities for biomaterial-based nanomedicine in the treatment of sepsis.

## Introduction

Sepsis is defined as a life-threatening organ dysfunction resulting from the host’s dysregulated response to infection [1]. Usually, it can be triggered by a wide variety of bacterial infections, leading to highly diverse clinical presentations. In sepsis, the host’s response to infection manifests with signs of infection accompanied by acute organ dysfunction. This dysfunction may lead to multiple organ failure, acidosis, and even death [2]. The pathogenesis of sepsis mainly encompasses pathogen invasion, dysregulated inflammatory-immune responses, oxidative stress, as well as tissue damage and subsequent organ failure induced by cell death. During the early stage, the invasion of pathogens triggers an excessive and uncontrolled inflammatory response, leading to cellular death and even tissue or organ damage. As inflammation transitions from an acute to a chronic phase, persistent depletion of immune cells drives the body into a state of immunoparalysis. In clinical settings, sepsis treatment has predominantly centered on managing the underlying disease, administering antibiotics, and implementing supportive care modalities. Initial treatment protocols usually involve incorporating early appropriate antimicrobial therapy, fluid resuscitation to restore tissue perfusion, and advanced interventions guided by ongoing assessments of resuscitation efficacy and organ dysfunction resolution [2,3]. Antibiotics constitute the cornerstone of pharmacotherapeutic interventions for sepsis. However, excessive utilization has expedited the proliferation of multidrug-resistant (MDR) pathogens colloquially termed “superbugs” [4]. It is noteworthy that the complex pathophysiology of sepsis, characterized by dynamic interactions among diverse toxins, inflammatory mediators, and cytokine storm processes across multiple signaling pathways, frequently necessitates comprehensive therapeutic strategies targeting multiple pathological components [5]. However, because of the dynamic nature of sepsis pathology, current regimens often fall short in achieving timely and coordinated modulation of both the hyperinflammatory and immunosuppressive phases. There is a critical need to explore more efficient strategies for the treatment of sepsis. The complex and dynamic pathophysiology of sepsis, characterized by an initial hyperinflammatory phase followed by a prolonged immunosuppressive state, demands therapeutic approaches that possess 2 essential characteristics: (a) multitarget synergistic action to address the diverse pathological drivers, and (b) temporal regulation capability to adapt to the shifting disease phases.

To address these issues, in recent years, with the rapid advancement of materials science and nanotechnology, interdisciplinary integration has offered new paradigms for disease treatment. Programmable biomaterial nanoplatforms, owing to their structural versatility, targeting precision, and stimulus-responsive behavior, have emerged as a particularly promising avenue for sepsis treatment [6]. For example, (a) the biomaterials serve as effective drug carriers by enabling controlled drug delivery and release, thereby enhancing therapeutic efficacy. During administration, these biomaterials can modulate specific features of the septic immune microenvironment either passively or actively by regulating oxidative stress, inflammation, and immune homeostasis, thereby establishing synergistic therapeutic approaches. They also provide an ideal platform for multitarget delivery or sequential administration of therapeutic agents [7]. (b) Beyond drug delivery, biomaterials, such as peptide nanofibers, demonstrate exceptional capabilities in capturing pathogens or modulating immune cell functions—including macrophage polarization, neutrophil activation and apoptosis, and the release of pro-inflammatory cytokines—thereby reversing the progression of sepsis. (c) Furthermore, in contrast to conventional pharmacotherapy, biomaterials augment the efficacy of therapeutic agents through enhanced biodistribution and bioavailability, thereby diminishing dependence on excessive antibiotic administration and curbing the emergence of antimicrobial resistance. Therefore, biomaterial-based nanomedicine may provide an alternative strategy for more efficient treatment of sepsis by enhancing targeted therapy efficiency, reducing the excessive use of antibiotics and enhancing their bioavailability, and enabling highly efficient regulation of the immune microenvironment.

Although some reviews have well summarized from the points of peptide-based nanomaterials for sepsis treatment [8], cell-membrane-coated nanoparticles for sepsis treatment [9], biomaterials for inflammation control [10], polymeric particle-based therapies for acute inflammatory diseases [11], and stimuli-responsive delivery systems for sepsis [12], a holistic viewpoint from different kinds of biomaterials in sepsis treatment, especially in immunomodulation, is still lacking. In this review, we provide a comprehensive review of the recent progress in biomaterial-based nanomedicine for sepsis treatment. First, we introduce the pathogenesis and pathological characteristics of sepsis. Then, the main treatment strategies of biomaterial-based nanomedicine were classified and discussed, particularly in the context of immune modulation. Finally, the existing challenges, gaps, and prospects in the future were also discussed.

## Pathogenesis and Pathological Characteristics of Sepsis

The pathophysiology of sepsis is characterized by infection-induced dysregulation of the systemic host response. The pathogenesis of sepsis primarily involves pathogen invasion, dysregulated inflammatory-immune responses, oxidative stress, mitochondrial dysfunction, cell death, and eventual tissue injury and organ failure. The host immune response is initiated upon pathogen invasion. Danger signals, such as damage-associated molecular patterns (DAMPs) or pathogen-associated molecular patterns (PAMPs), are released from damaged cells or pathogens. These signals activate resident immune cells, prompting them to generate cytokines, chemokines, and oxygen-free radicals [13]. Subsequently, circulating immune cells adhere to endothelial cells and migrate to the sites of inflammation, thereby amplifying the pro-inflammatory response to destroy pathogens [14]. This coordinated immune response is intended to restore immune homeostasis. However, an inadequate or excessive reaction can initiate a disastrous cascade, marked by local or systemic tissue damage and an overproduction of danger signals. Moreover, the transition from acute to chronic inflammatory responses in sepsis may also precipitate a collapse of immune tolerance, ultimately leading to immunoparalysis, recurrent infections with drug-resistant bacteria, and potentially death [15]. Hence, immune homeostasis holds a pivotal position in the pathophysiology of sepsis and substantially influences clinical outcomes.

## Pathogen Invasion and Dysregulated Inflammatory-Immune Response: The Initiation Point of Sepsis

The immune response in sepsis is initiated following pathogen invasion, wherein immune cells detect PAMPs or DAMPs via Toll-like receptors (TLRs), nucleotide-binding oligomerization domain (NOD)-like receptors (NLRs), and other pattern recognition receptors (PRRs), subsequently activating inflammatory signaling pathways [16]. This activation triggers the engagement of adaptor proteins, such as myeloid differentiation primary response 88 (MyD88), and pro-inflammatory transcription factors, exemplified by nuclear factor-κB (NF-κB) [17]. As a result, a plethora of inflammatory molecules are secreted, including interleukin-1α (IL-1α), interleukin-1β (IL-1β), interleukin-6 (IL-6), interleukin-8 (IL-8), tumor necrosis factor-alpha (TNF-α), and high mobility group box 1 (HMGB1), all of which exhibit significant up-regulation [18,19]. Additionally, there is an increased release of inflammatory mediators, such as monocyte chemoattractant protein 1, phosphatidylinositol, glutamate, reactive oxygen species (ROS), and nitric oxide (NO) [20,21]. Concurrently, in response to cytokines, PAMPs, and DAMPs, endothelial cells up-regulate the expression of adhesion molecules, including E-selectin and intercellular adhesion molecule-1 (ICAM-1), thereby facilitating the recruitment of leukocytes to inflamed sites. The breakdown of endothelial barrier integrity permits the infiltration of various cytokines and leukocytes into tissues, subsequently inducing parenchymal cell apoptosis and dysfunction, thereby aggravating the progression of sepsis [22–24]. Activated endothelial cells not only compromise the integrity of the vascular barrier but also modify microcirculation and vascular tone, ultimately leading to impaired blood perfusion. These alterations can give rise to ischemic or hemorrhagic lesions [25,26].

Studies indicate that pro-inflammatory responses and immunosuppression occur concurrently, with their magnitude being modulated by multiple host factors (e.g., genetic background and comorbidities) as well as pathogen factors (e.g., strain type, virulence, and infectious load) [27–29]. Sepsis-associated immunosuppression arises from impairments in both innate and adaptive immunity. Mechanisms driving innate immune dysregulation include defective neutrophil recruitment and trafficking, aberrant macrophage differentiation and polarization, suppressed dendritic cell immune function, impaired natural killer (NK) cell cytotoxicity and cytokine production, and excessive activation of the complement system. In the adaptive immune system, characteristic dysfunctions encompass T cell dysfunction and numerical depletion, elevated frequency of regulatory T cells (Tregs), imbalanced Th17/Treg ratio, impaired B cell function, and reduced immunoglobulin levels [30]. During immunosuppression, T cell numbers decline drastically as a result of enhanced apoptosis and up-regulated expression of inhibitory immune checkpoint molecules, which further exacerbates immunosuppression and may even culminate in immune collapse [31,32]. Inflammation-associated immunosuppression represents a key determinant of secondary infections and multiple organ dysfunction syndrome, which are major contributors to adverse outcomes in patients with sepsis [28,33].

## Oxidative Stress and Mitochondrial Dysfunction: Amplifiers of Injury

Oxidative stress and mitochondrial injury are pivotal mechanisms in sepsis; these processes can elicit inflammatory responses and generate deleterious oxidative products, such as reactive nitrogen species, which exert detrimental effects on cells. Specifically, mitochondrial responses to inflammatory mediators, ROS, and NO lead to diminished adenosine triphosphate (ATP) production, with suppressed mitochondrial electron transport chain activity and oxidative phosphorylation culminating in mitochondrial dysfunction, which in turn triggers bioenergetic perturbation and cellular functional impairment [34]. Notably, during inflammation, ROS and nitric oxide synthase (NOS) can activate Toll-like receptor 4 (TLR4)-mediated NF-κB and interferon regulatory factor signaling pathways, which play a prominent role in driving apoptosis and endothelial vasculopathy [35,36]. Dysregulated oxidative stress perturbs cellular respiration and metabolism, thereby promoting further ROS generation; this subsequently induces lipid, DNA, and protein damage, coupled with energy exhaustion, ultimately culminating in cell death and secondary organ injury [37].

Furthermore, in the pathogenesis of sepsis, tissue hypoperfusion and microcirculatory dysfunction frequently lead to localized or systemic hypoxia, which severely compromises mitochondrial oxidative phosphorylation and ATP generation. During the progression of sepsis, the resultant depletion of ATP similarly impairs energy-dependent cellular processes, triggering a cascade of deleterious events. For example, the failure of Na^+^/K^+^-ATPase pumps due to energy deprivation disrupts ionic homeostasis, leading to elevated intracellular sodium and calcium concentrations, collapse of the electrochemical gradient, and subsequent cytotoxic edema. These disturbances, in turn, exacerbate mitochondrial dysfunction, activate calcium-dependent proteases (e.g., calpains), promote the accumulation of ROS, and stimulate excessive NO production. Collectively, these interconnected mechanisms amplify oxidative stress, propagate inflammatory signaling, and ultimately induce a cascade of programmed cell death pathways, thereby contributing to the progression of sepsis-induced tissue injury and multiorgan failure [38].

## Programmed Cell Death: A Double-Edged Sword in Sepsis Pathophysiology

Programmed cell death is an active process of cellular clearance triggered by specific signals or stimuli that maintains tissue homeostasis under physiological conditions; apoptosis constitutes a key mechanism in sepsis pathogenesis, mediating cell death that is often accompanied by concurrent autophagy. In the cecal ligation and puncture (CLP) mouse model, the expression levels of cytochrome c, caspase-3, caspase-8, and caspase-9 were markedly up-regulated, whereas that of Bcl-2 was suppressed, thereby collectively promoting immune cell apoptosis [39]. Beyond apoptosis, pyroptosis has emerged as another form of programmed cell death characterized by progressive cell swelling culminating in plasma membrane rupture, which facilitates the release of intracellular contents and triggers a robust inflammatory cascade [40]; this process is primarily mediated by caspase-dependent activation of the gasdermin (GSDM) protein family. Macrophage pyroptosis can be elicited via diverse mechanisms, leading to the release of copious amounts of inflammatory mediators that profoundly exacerbate inflammatory responses and organ dysfunction.

Ample evidence confirms that autophagy serves as a critical regulator of inflammatory responses [41,42]; autophagy plays a key role in modulating macrophage activation, with inadequate autophagy potentially driving excessive macrophage activation and polarization, thereby exacerbating inflammatory responses [43], whereas enhanced autophagy typically exerts anti-inflammatory effects. Owing to its homeostatic functions, autophagy has been shown to safeguard the immune system by eliminating pathogens, stabilizing mitochondrial membranes, and preventing immune cell apoptosis [44,45]. Conversely, inhibiting autophagy can enhance the antimicrobial capacity of macrophages [46]. Although autophagy activates neutrophils and contributes to the formation of neutrophil extracellular traps (NETs) [47], it can also mitigate inflammation and mediate tolerance to toxins such as *Staphylococcus aureus* alpha-toxin [48]. These findings collectively suggest that autophagy may modulate the release of inflammatory mediators and contribute to a state of relative immune tolerance.

During inflammation, activated neutrophils undergo a process known as “NETosis”, in which they flatten and form prominent extracellular structures called NETs—a distinct form of cell death [49]. NETs are web-like structures made up of antimicrobial proteins such as serine proteases, cathepsin G, proteinase 3, and neutrophil elastase (NE) and the neutrophil’s own DNA. When depolymerized chromatin and intracellular granular proteins are released, neutrophils activate, capture, and eliminate pathogens. This process, known as NETosis, would cause neutrophils to perish. In addition to effectively eradicating bacteria, the fibrous architecture of NETs can act as a physical barrier to prevent bacterial dissemination. On the other hand, the immune system may be compromised by excessive exposure to extracellular histone complexes, indicating that NETosis represents a double-edged sword for immunity [50].

## Tissue and Organ Damage: The Consequence of Sepsis-Induced Injury

**Fig. 1.**
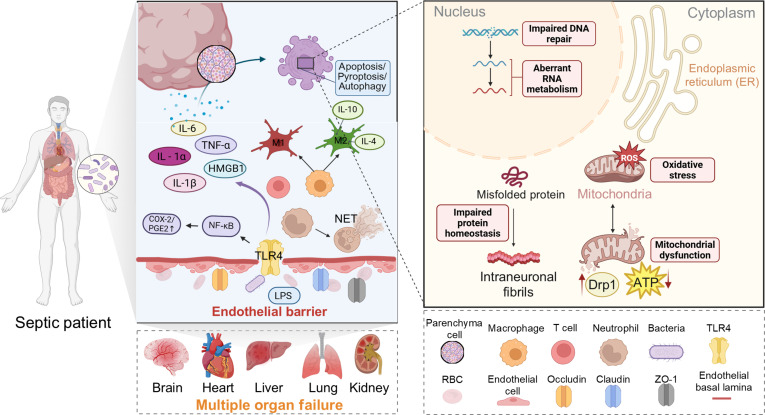
Pathophysiological mechanism of sepsis. Upon infection, pathogen recognition triggers an initial immune response and cytokine storm. Failure to clear pathogens leads to sustained inflammation, resulting in tissue/organ damage.

The development and progression of sepsis-associated organ dysfunction involves not only systemic, life-threatening pathophysiological processes but also organ-specific injury mechanisms. Tissue hypoxia, mitochondrial dysfunction, and apoptosis are all well-recognized key mechanisms underlying sepsis-induced organ dysfunction [51]. In sepsis, the host’s response to infection is characterized by the concurrent presence of signs of infection and acute organ dysfunction. Sepsis typically perturbs blood flow distribution to organ systems via vasodilation and microcirculatory dysfunction [52]; increased endothelial barrier permeability drives fluid extravasation, resulting in tissue edema and organ failure [53]. Pro-inflammatory mediators trigger the coagulation cascade, leading to a hypercoagulable state that manifests as disseminated intravascular coagulation (DIC), which exacerbates organ dysfunction by compromising blood flow and oxygen delivery [54,55]. Studies have demonstrated that heparin provides endothelial cell protection by inhibiting heparinase, ameliorating DIC, and thereby attenuating tissue and organ damage in sepsis [56]. Furthermore, mitochondrial dysfunction can induce cytopathic hypoxia, a condition in which tissues are unable to efficiently utilize oxygen despite adequate oxygen supply [57]. Accordingly, organ dysfunction serves as a critical indicator for predicting patient prognosis, with multiple organ dysfunction being closely correlated with a high mortality risk [2]. The consequences are particularly serious when acute injury involves key organs such as the brain, cardiovascular system, kidneys, liver, and lungs. Such dysfunction may lead to multiple organ failure, acidosis, and even death [2]. The pathophysiological mechanisms of sepsis are illustrated in Fig. 1. The major pathogenesis and pathological characteristics of sepsis are shown in Table 1.

**Table 1.** Major pathophysiological mechanisms and pathological characteristics of sepsis

Pathophysiological mechanism	Key mediators/cellular events	Pathological characteristics	Ref.
Dysregulated inflammatory-immune response	PAMPs/DAMPs → TLR/NLR activation → NF-κB, MyD88↑ → IL-1β, IL-6, TNF-α, ROS, HMGB1↑	Cytokine storm; endothelial cell activation; leukocyte infiltration	[16–18]
Oxidative stress and mitochondrial dysfunction	ROS, NO, iNOS↑ → mitochondrial electron transport chain inhibition → ATP depletion	Energy metabolism dysfunction	[32–35]
Programmed cell death	Caspase-3/8/9 activation →apoptosis; Caspase-1/4/5/11 activation →GSDMD cleavage→pyroptosis; ROS↑ → chromatin decondensation and neutrophil extracellular traps release → NETosis	Lymphocyte depletion; organ dysfunction; inflammatory signaling amplification	[36–47]
Endothelial barrier disruption	ICAM-1, VCAM-1, E-selectin↑ → endothelial barrier integrity breakdown	Leukocyte infiltration; widespread coagulation activation; microthrombi formation; microcirculatory dysfunction	[21–25]
Immunosuppression/Immunoparalysis	T cell apoptosis↑, Treg↑, B cell function↓, monocyte↓	Immune cell dysfunction; immunoglobulin level reduction	[26–31]

PAMPs, pathogen-associated molecular patterns; DAMPs, damage-associated molecular patterns; TLR, Toll-like receptor; NLR, NOD-like receptor; NF-κB, nuclear factor-κB; MyD88, myeloid differentiation primary response 88; IL-1β, interleukin-1β; IL-6, interleukin-6; TNF-α, tumor necrosis factor-alpha; ROS, reactive oxygen species; HMGB1, high mobility group box 1; NO, nitric oxide; iNOS, inducible nitric oxide synthase; GSDMD, gasdermin D; ICAM-1, intercellular adhesion molecule-1; VCAM-1, vascular cell adhesion molecule-1; Treg, regulatory T cell

## Emerging Nanomedicine Strategies for Sepsis

### Classification of biomaterials for sepsis treatment

**Fig. 2.**
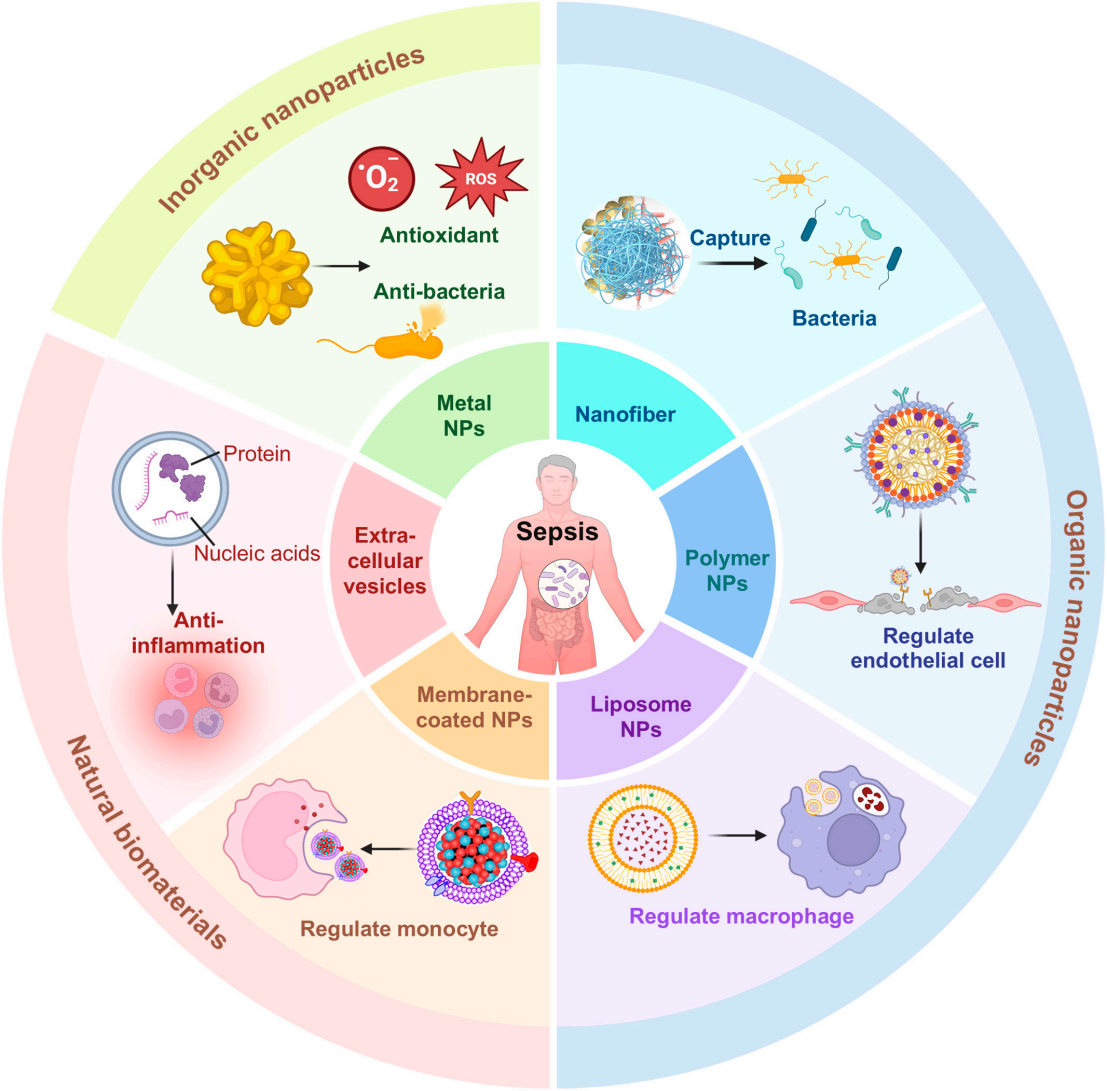
Overview of biomaterial-based nanomedicine for sepsis treatment. This schematic illustrates the primary categories of biomaterials, encompassing organic, inorganic, and natural subtypes, along with their critical roles in modulating immune responses and eliminating pathogens during sepsis management.

For sepsis treatment, biomaterials are usually classified into 3 categories: organic biomaterials (including nanofibers, polymeric nanoparticles, and liposomal nanoparticles), inorganic biomaterials, and natural biomaterials (including extracellular vesicle [EV]-based nanoparticles, cell membrane-coated nanoparticles, and cell therapies) (Fig. 2). For example, nanofibers demonstrate remarkable bioadhesive properties, enabling them to effectively capture pathogens present in the bloodstream, thereby playing a pivotal role in controlling the progression of both infection and inflammation [58]. Polymer nanoparticles possess significant diversity in molecular weight and structural configuration, which enables efficient encapsulation of diverse therapeutic agents while facilitating straightforward surface functionalization for controlled-release formulations and targeted delivery applications [59]. As such, various surface receptors have been utilized to design receptor-targeted polymer nanodrug delivery systems. For instance, surface modification with targeted ICAM-1 on the nanoparticle surface, which is overexpressed on activated endothelial cells, can effectively deliver drugs to alleviate endothelial barrier damage [60]. Liposomes are extensively employed for the delivery of small molecules, peptides, proteins, genes, and antibodies, owing to their high drug-loading capacity, low toxicity, targeting ability, biocompatibility, biodegradability, and optimized pharmacokinetics [61]. Recently, biomimetic cell membrane-coated nanoparticles have demonstrated exceptional bio-camouflage capabilities, enabling evasion of immune recognition, precise targeting of inflammatory sites, and efficient traversal of biological barriers [62]. These nanoparticles retain the antigenic surface characteristics of their source cells, enabling them to mimic cellular functions and effectively modulate the immune response. EVs are lipid bilayer-enclosed vesicles released by cells under physiological and pathological conditions [63]. These structures contain bioactive molecular cargo, including proteins, nucleic acids, lipids, and metabolites, thereby facilitating intercellular communication through their mediator functions. Numerous studies have employed bioactives derived from EVs or encapsulated other bioactives to modulate the inflammatory progression associated with sepsis. Moreover, inorganic nanoparticles possess high stability, large specific surface areas, and tunable surface chemistries, making them ideally suited for drug loading and photothermal therapy applications. These characteristics collectively make them a promising therapeutic platform with inherent antioxidant and bactericidal properties [64].

**Fig. 3.**
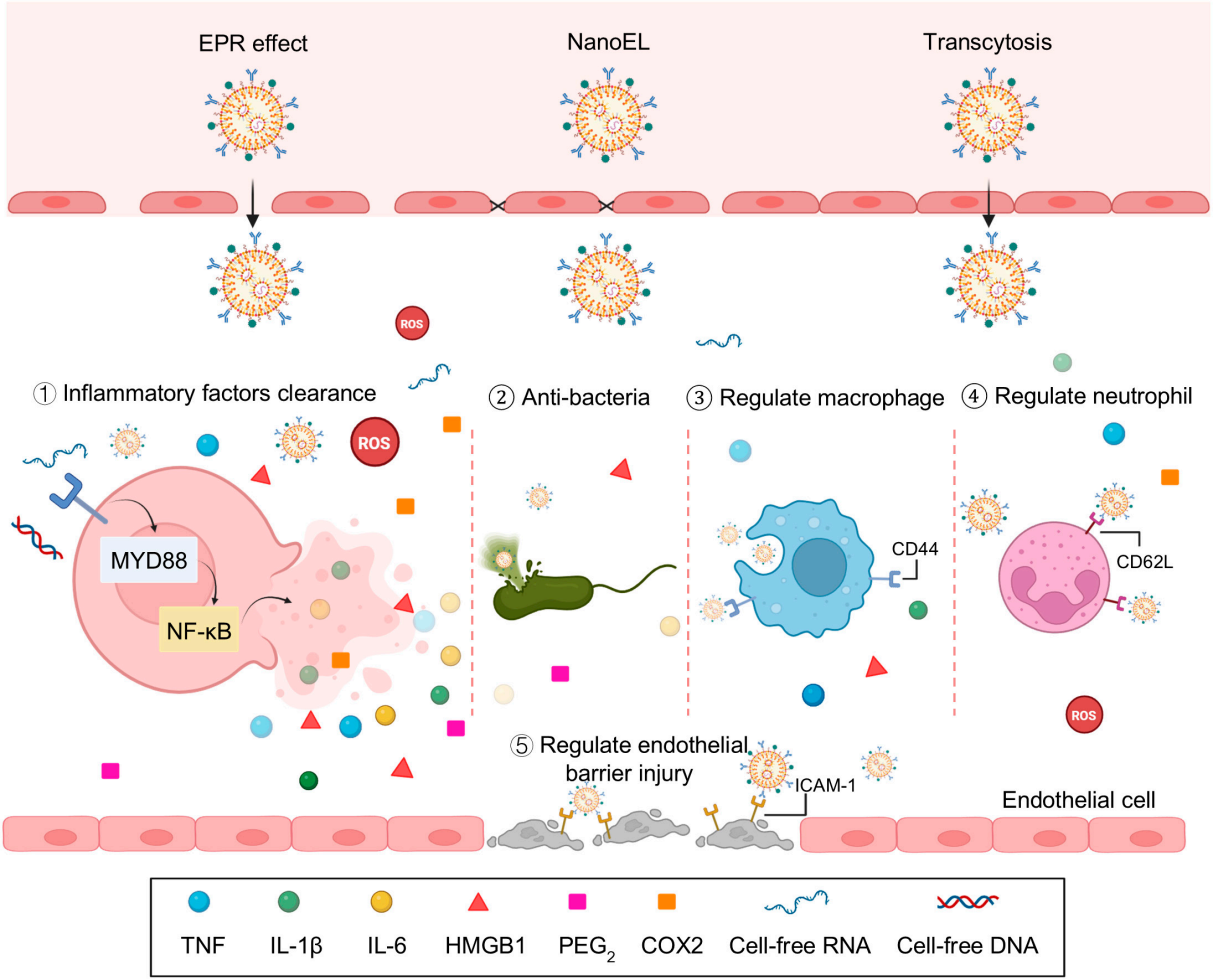
Biomaterial-based nanomedicine for the regulation of sepsis microenvironment. Biomaterial-based nanomedicines are usually delivered into the inflammatory microenvironment through various pathways, such as transcytosis, the enhanced permeability and retention (EPR) effect, and nanomaterial-induced endothelial leakage (NanoEL). These delivery strategies enable them to achieve highly efficient clearance of inflammatory factors and pro-inflammatory mediators from the microenvironment. They can also eliminate pathogens, target modulation of immune cell function (e.g., macrophage and neutrophil), and alleviate vascular endothelial barrier injury to recover immunological homeostasis.

Conventional sepsis treatment is usually constrained by nonspecific drug distribution, systemic toxicity, and limited immunomodulatory capacity. Compared to conventional drug therapies, biomaterial-based nanomedicine exhibits several advantages, including targeted drug delivery in vivo, minimized drug wastage, enhanced therapeutic efficacy, reduced required dosages, and diminished biological toxicity [65,66]. These biomaterials are typically administered into the inflammatory microenvironment via multiple transport mechanisms, including transcytosis, the enhanced permeability and retention effect, and nanomaterial-induced endothelial leakage. When they are delivered into the injured tissues, they can efficiently remove excessive inflammatory factors and pro-inflammatory mediators from the microenvironment. Additionally, these systems can be designed to specifically modulate immune cell functions, eliminate pathogens, and modulate endothelial cell activation, thereby restoring immunological homeostasis (Fig. 3). In summary, through diversified design and functional modifications, nanomaterials address the limitations of conventional drug delivery systems, enabling precise modulation of the immune microenvironment in sepsis and offering potential for temporally and spatially controlled therapeutic strategies.

### Nanomedicine for endotoxin neutralization and pathogen capture in sepsis

#### Endotoxin neutralization strategies

Endotoxin, also termed lipopolysaccharide (LPS), constitutes an essential component of the outer membrane in gram-negative bacteria and represents one of the most critical PAMPs in sepsis [67]. As the most potent pyrogen, exposure to even minute quantities of endotoxin can induce clinical manifestations [68]. As a core trigger of the inflammatory cascade in sepsis, endotoxin plays a pivotal role in disease progression, making its direct removal from circulation crucial for blocking sepsis advancement. While conventional blood purification techniques show limited efficiency and are prone to side effects, functional nanomaterials offer a novel approach for efficient and safe endotoxin clearance through specific adsorption and neutralization mechanisms. The elimination of endotoxin via extracorporeal approaches, particularly hemoperfusion, constitutes a promising research focus [69]. In aqueous solutions, owing to its amphiphilic nature, endotoxin can aggregate into supramolecular assemblies. The phosphate groups of monomeric endotoxin form the outer layer of these assemblies, while the remaining parts constitute the inner layer. This particular structure enables endotoxin aggregates to interact with cationic adsorbents [70].

Polymyxin B (PMB), a cationic antimicrobial peptide (AMP), can bind and neutralize endotoxin [71]. However, its nephrotoxicity and neurotoxicity have limited the clinical use of PMB at systemically effective doses for sepsis therapy [72]. To reduce the cytotoxicity of PMB against mammalian cells while preserving its therapeutic efficacy, Yuk et al. [73] engineered a multifunctional nanoparticle designated as D-TZP (nanoparticle containing vitamin D, tannic acid, LMZWC, and PMB). D-TZP is fabricated by an iron-complexed tannic acid (TA) nanocapsule containing a vitamin D core, coated with PMB and low-molecular-weight zwitterionic chitosan (LMZWC). Within the PMB, LMZWC appears to function as a “bumper” layer to prevent direct contact of PMB with mammalian cells, thereby reducing membrane damage; vitamin D exerts efficient immunomodulatory activity by down-regulating TLR2/4 synthesis and antagonizing the effect of LPS on proinflammatory cytokine production. Collectively, D-TZP attenuates the membrane toxicity of PMB while preserving its endotoxin-neutralizing capacity.

In addition to endotoxin, cell-free DNA (cfDNA) also significantly contributes to sepsis progression. cfDNA enters the systemic circulation following cellular injury, death (including necrosis), or infection, subsequently initiating inflammatory cascades through the activation of PRRs such as TLRs [74]. Li et al. engineered a multifunctional nanoparticle (designated TMP, nanoparticle containing tannic acid, manganese, and polymyxin B) for the clearance of multiple inflammatory mediators, including LPS, ROS, and cfDNA. The TMP is fabricated by the TA, PMB, and manganese (Mn) via hydrogen bonding and coordination interactions. Within the TMP, PMB enables efficient adsorption of LPS, while manganese serves to scavenge ROS and enhance the stability of the material; meanwhile, TA interacts with the phosphate backbone of DNA through hydrogen bonding, forming stable TA–DNA complexes that facilitate cfDNA removal [75]. This highlights the capability of nanomaterials for targeted optimization in sepsis toxin-clearance therapies.

#### Pathogen capture and clearance strategies

**Fig. 4.**
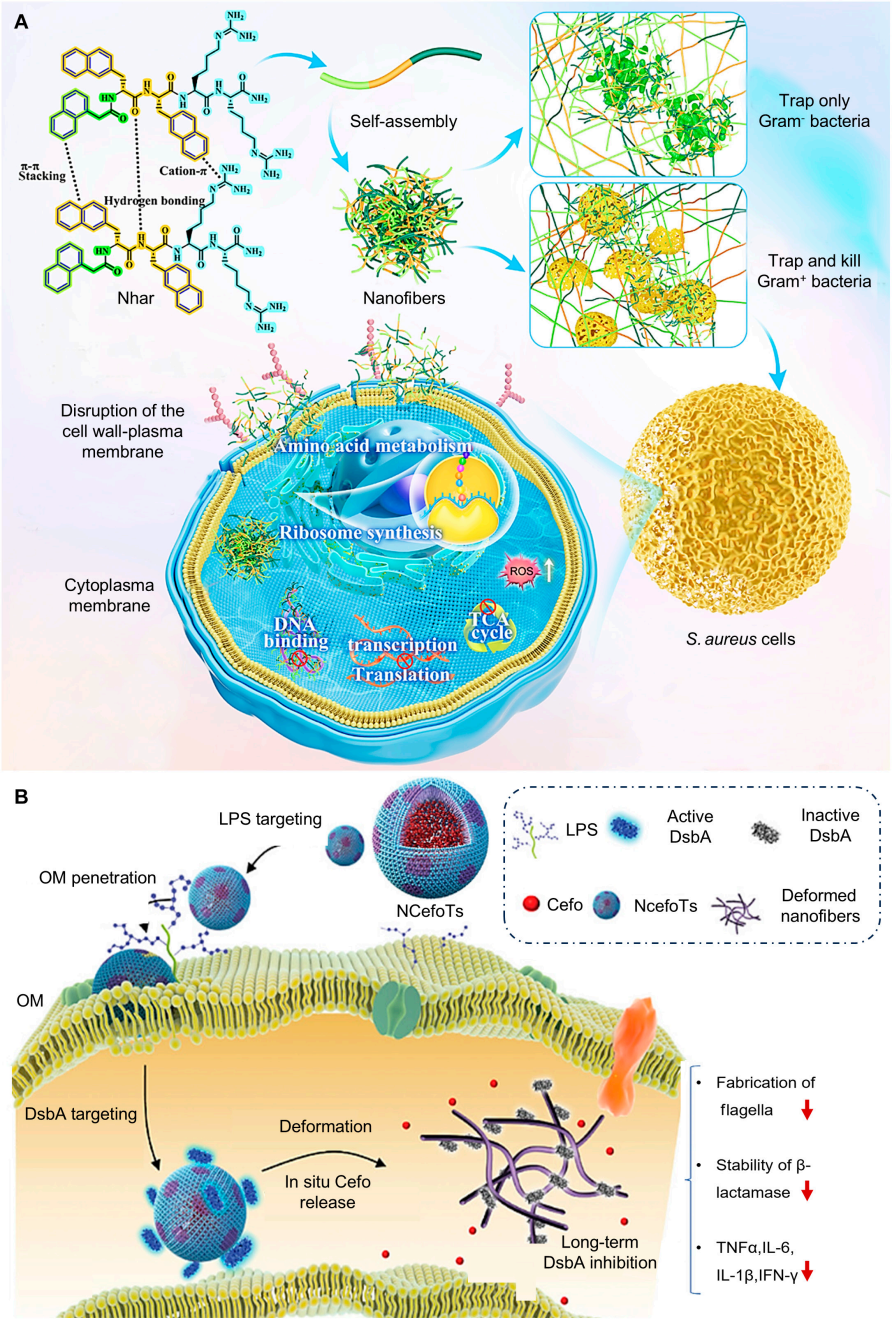
Nanofiber-based pathogen capture and clearance strategies. (A) Schematic of the self-assembled peptide nanofibrous matrices (Nhar) for pathogen capture. Nhar enables them to self-assemble into nanofibers that trap and kill circulating bacteria through membrane disruption and inhibition of essential metabolic processes. Reprinted with permission from Ref. [58]. Copyright 2025, *Sci. Adv*. (B) Schematic illustration of the mechanisms employed by NCefoTs against MDR *E. coli*: initial penetration through the OM surface via LPS-targeted MLp; subsequent inhibition of DsbA through enzymatic tailoring and in situ nanofibril deformation; final synergistic bactericidal effect achieved through the inactivation of DsbA and intracellular delivery of Cefo. Reprinted with permission from Ref. [79]. Copyright 2024, *Adv. Mater*.

The growing global concern over antibiotic resistance highlights the urgent need for effective antimicrobial agents. The application of nanofibrous matrices (such as AMP nanofibers) for bacterial capture has recently emerged as a novel antimicrobial approach, garnering significant scientific interest [76,77]. For example, AMPs combat resistant bacteria by disrupting membrane structures. AMPs are predominantly secreted by neutrophils and epithelial cells and function as a front-line defense against pathogenic assaults [78]. Yu et al. engineered a dual-functional AMP nanofiber system (Nhar) for capturing pathogens and restricting their dissemination. The Nhar is fabricated by a self-assembly of hydrophobic (h) and aromatic (ar) interactions [58]. Nhar enables efficient membrane transport inhibition through electrostatic binding to negatively charged bacterial components, which compromises membrane fluidity and permeability in gram-positive bacteria; after penetrating the bacterial cell, Nhar further inhibits energy metabolism, transcription, translation processes, and ribosome synthesis, ultimately leading to bacterial death (Fig. 4A). However, because of the distinct outer membrane architecture of gram-negative bacteria containing LPS, Nhar only achieves physical entrapment without penetrating the inner membrane to induce intracellular damage. In a mouse bacteremia model induced by *S. aureus*, Nhar exhibits significant therapeutic efficacy.

The issue of antibiotic resistance is increasingly severe; conventional antimicrobial agents fall short in addressing infections caused by MDR bacteria. Disulfide bond (Dsb) proteins play a crucial role in the oxidative protein folding pathway within the periplasm of gram-negative bacteria, promoting the folding and stability of numerous proteins. They represent a promising yet underexploited target for combating MDR bacteria, as precise subcellular targeting through multiple bacterial barriers remains a major challenge. To achieve targeted inhibition of DsbA, Zou et al. [79] engineered a novel heterotypic phase-separated nano-antibiotic termed NCefoTs for combating pathogens through physical capture and bactericidal action. The NCefoTs is fabricated by an enzyme-inhibiting lipopeptide (ELp component), a membrane-recognizing and disrupting lipopeptide (MLp component), and the antibiotic cefoperazone. Within the NCefoTs, MLp contains an LPS-targeting sequence, a membrane-disruptive peptide sequence, and a DsbA-cleavable sequence, which promotes efficient penetration of the nano-antibiotics across the bacterial outer membrane (OM); simultaneously, ELp specifically targets and inhibits DsbA via DsbA recognition, enzymatic modulation, and in situ nanofiber transformation. Ultimately, synergistic bactericidal effects are achieved through the inactivation of DsbA and the intracellular delivery of cefoperazone (Fig. 4B). This establishes a novel therapeutic platform that integrates structural biomimicry with functional synergy for pathogen clearance in the early stage of sepsis, offering a promising strategy against drug-resistant bacterial infections.

**Fig. 5.**
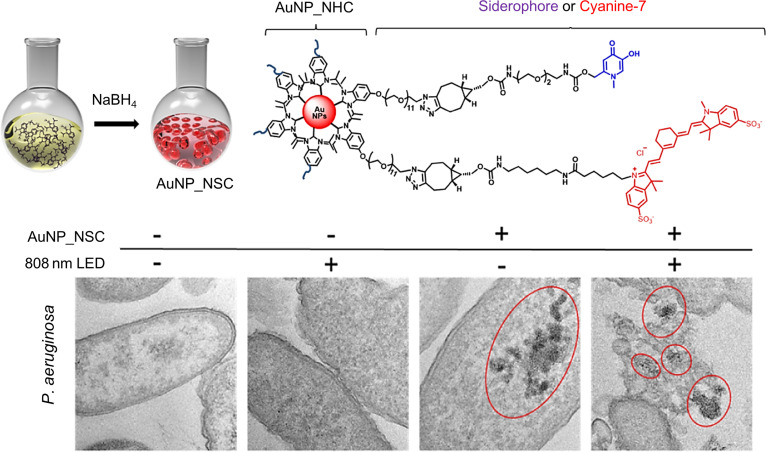
Functional siderophore-functionalized gold nanoparticles (AuNP-NSC) for the targeted elimination of antibiotic-resistant *Pseudomonas aeruginosa*. Reprinted with permission from Ref. [80]. Copyright 2025, *ACS Nano*.

Metal nanoparticle-based platforms can be integrated with multiple therapeutic strategies, providing more effective treatment alternatives against MDR bacteria. For instance, gold nanoparticles (AuNPs) and silver nanoparticles (AgNPs) have been shown to possess significant anti-inflammatory properties. However, their application is limited by poor stability, low drug-loading capacity, and difficulties in controlling drug release. Ha et al. [80] engineered an AuNP-based construct termed AuNP-NSC (Gold nanoparticle_N-heterocyclic_Siderophore_Cyanine7) for disrupting MDR bacterial cells. The AuNP-NSC is fabricated by Au nanoparticles and N-heterocyclic carbenes (NHCs). Within the AuNP-NSC, the introduced NHC exhibits strong affinity for transition metals to form highly stable complexes with AuNPs via covalent bonding. AuNP-NSC specifically targets and enters drug-resistant gram-negative *Pseudomonas aeruginosa* by binding trivalent iron, achieving selective localization. Upon exposure to near-infrared (NIR) light, the AuNPs generate localized heat and mechanical vibrations, thereby inducing cytotoxicity in adjacent bacterial cells (Fig. 5). Treatment with AuNP-NSC significantly inhibited the proliferation of antibiotic-resistant *P. aeruginosa* and mitigated drug resistance, markedly preventing systemic organ failure in sepsis mouse models. This exemplifies the potential of rationally designed nanomaterials to achieve enhanced bactericidal efficacy in sepsis pathogen-clearance therapies.

### Nanomedicine for endothelial repair in sepsis

**Fig. 6.**
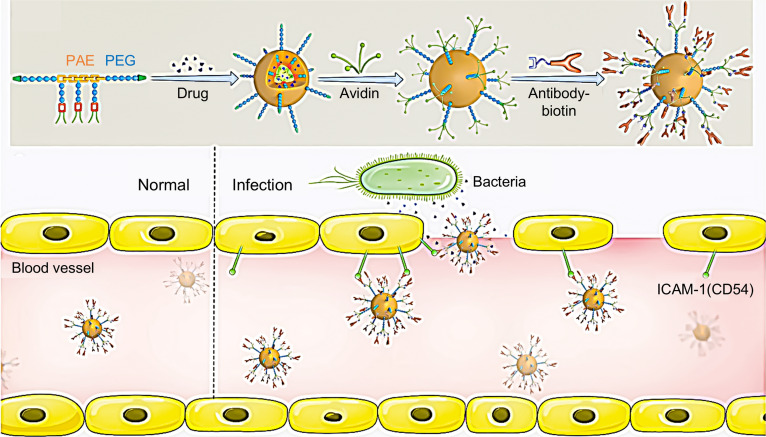
Schematic illustration of pH and enzyme-responsive polymer micelle drugs for sepsis treatment. Within the IME, these polymer micelles demonstrate pH- and enzyme-responsive properties, facilitating controlled release of the antibiotic and anti-inflammatory agents, thereby significantly reducing bacterial burden and modulating the host inflammatory response. Reprinted with permission from Ref. [60]. Copyright 2018, *Adv Mater*.

In sepsis, endothelial cell activation and barrier disruption play a pivotal role in organ injury. Conventional anti-inflammatory and antibiotic therapies often lack specific targeting toward damaged endothelium. LPS-activated human umbilical vein endothelial cells (HUVECs) display a high-level expression of specific surface antigens, encompassing ICAM-1, vascular cell adhesion molecule-1 (VCAM-1), and selectins. Functionalized nanoparticles can recognize activated endothelial surface markers such as ICAM-1, enabling drug enrichment and controlled release at the site of infection, thereby synergistically exerting anti-inflammatory and antibacterial effects. Zhang et al. [60] engineered amphiphilic chimeric copolymers that self-assemble into polymeric micelles to achieve targeted drug delivery at infection sites. Polymer micelles are constructed via the self-assembly of pH-sensitive copolymers incorporating bacterial enzymes, forming micellar structures coencapsulating the antibiotic ciprofloxacin and the anti-inflammatory agent TPCA-1 (2-[(aminocarbonyl)amino]-5-(4-fluorophenyl)-3-thiophenecarboxamide), followed by conjugation with ICAM-1 antibodies for targeted delivery to infectious microenvironments (IMEs) (Fig. 6). Within the IME, these polymer micelles exhibit excellent pH and enzyme-responsive properties, enabling them to controllably release the antibiotic and the anti-inflammatory agents, significantly reducing the bacterial burden and modulating the host inflammatory response. Consequently, the treatment effectively alleviated the inflammatory responses while re-establishing vascular homeostasis in septic mice.

The bacterial pathogen *S. aureus* stands as a principal culprit in bloodstream infections, with patients frequently suffering from recurrence even after undergoing antibiotic therapy. The vascular endothelium serves as a primary target in sepsis-related pathophysiological events, and a multitude of pathogens, *S. aureus* included, have been demonstrated to bind to and internalize into human vascular endothelial cells [81,82]. Conventional anti-staphylococcal agents exhibit restricted efficacy against internalized bacteria. However, nanoparticles conjugated with antibiotics can surmount these challenges. Nader et al. [83] engineered the vancomycin hydrochloride (Vh)-encapsulated poly(lactic-co-glycolic acid) (PLGA) nanoparticles to suppress bacterial growth within infected endothelial cells. The Vh-PLGA nanoparticles are internalized efficiently via PLGA-mediated endocytosis. Following endosome–lysosome fusion, the acidic intraluminal environment triggers nanoparticle degradation, leading to the direct intracellular release of vancomycin and thereby eliminating internalized bacteria. Thus, this endothelium-targeted nano-delivery system not only enhances local drug concentration at the infection site but also achieves dual therapeutic efficacy—combining bacterial clearance with endothelial protection—through a microenvironment-responsive release mechanism, which significantly improves outcomes in sepsis.

### Nanomedicine for macrophage immunomodulation in sepsis

#### Organic biomaterials-mediated macrophage modulation strategies

**Fig. 7.**
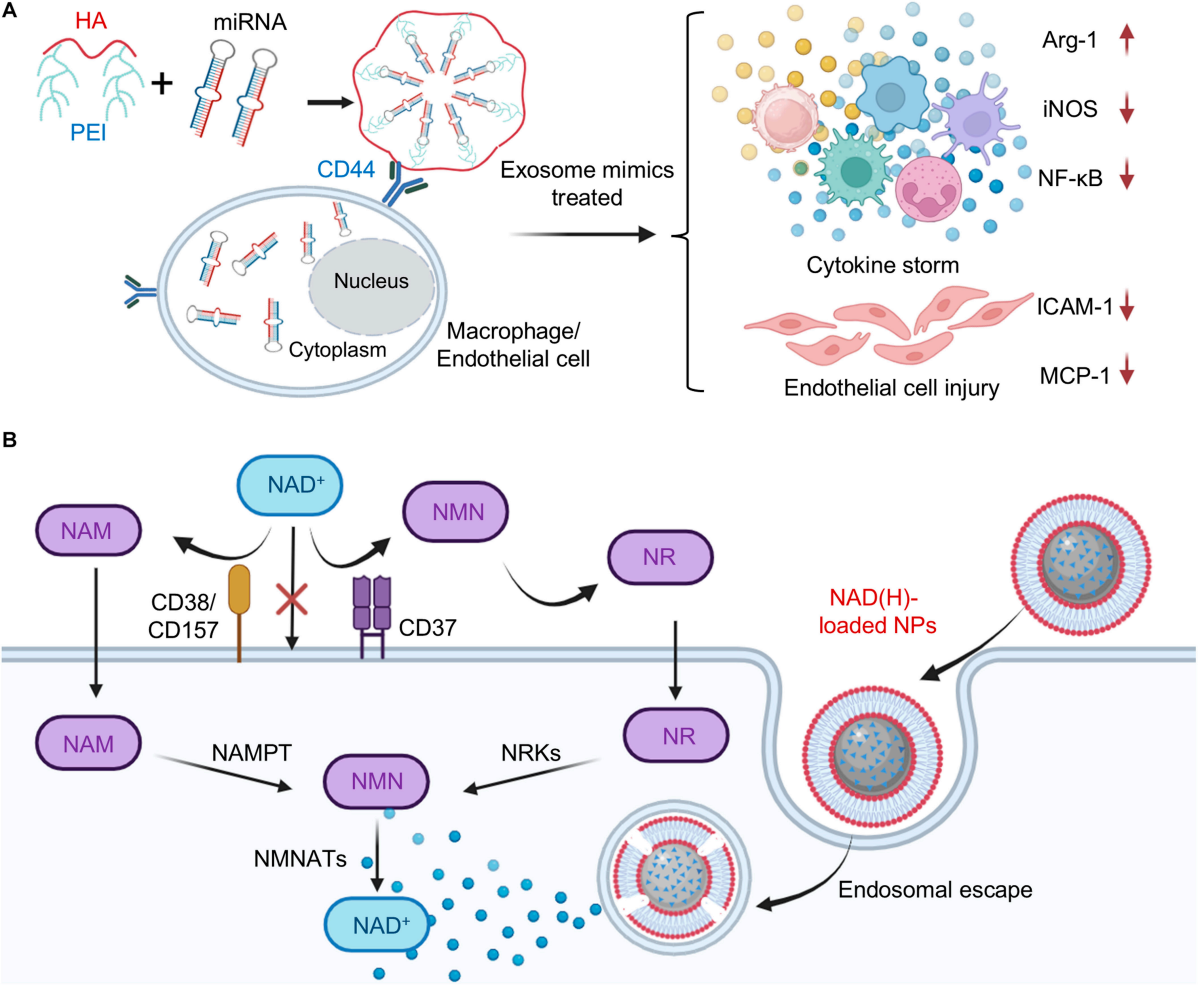
Organic biomaterial-based macrophage modulation strategies for sepsis treatment. (A) Schematic diagram of the synthetic exosome mimics for sepsis treatment. The exosome mimics are fabricated by the self-assembly of 7 anti-inflammatory miRNAs from melanoma-derived exosomes, with a hyaluronic acid (HA)–polyethyleneimine (PEI) copolymer. They can target CD44 on macrophages to alleviate the inflammation storm in sepsis. Reprinted with permission from Ref. [85]. Copyright 2022, *Adv. Mater*. (B) Schematic illustration of liposome-coated CaP or MOF nanoparticles for NAD(H) delivery and sepsis microenvironment modulation. Reprinted with permission from Ref. [86]. Copyright 2022, *Nat. Nanotechnol*.

Intervention targeting the hyperactivation of immune cells represents a promising therapeutic strategy for sepsis. Macrophages play a central role in the immune dysregulation of sepsis, where their excessive activation drives the cytokine storm [84]. Several studies suggest that immunosuppressive tumors may attenuate sepsis-associated immune hyperactivation, thereby alleviating sepsis. Li et al. [85] performed transcriptomic sequencing and identified that melanoma-derived exosomes exert good anti-inflammatory effects primarily through 7 specific miRNAs. Based on this result, they developed a biomimetic exosome mimic, which is fabricated by the self-assembly of 7 anti-inflammatory miRNAs of melanoma-derived exosomes and a hyaluronic acid (HA)–polyethyleneimine (PEI) copolymer (Fig. 7A). Within the exosome mimic, HA can target both macrophages and activated vascular endothelial cells via the CD44 antigen, enabling targeted delivery of the anti-inflammatory miRNAs. The delivered miRNAs significantly up-regulated the anti-inflammatory M2 marker Arg1 expression in macrophages and down-regulated pro-inflammatory M1 markers inducible nitric oxide synthase (iNOS) and the transcription factor NF-κB, thereby promoting macrophage polarization toward an anti-inflammatory phenotype. Moreover, they could also suppress the expression of adhesion molecules (ICAM-1 and MCP-1) in injured vascular endothelial cells. As a result, administration of these exosome mimics markedly reduced serum levels of IL-6, IL-8, and TNF-α, alleviated clinical symptoms in both septic mice and cynomolgus monkeys, and ultimately led to a significant improvement in survival rates.

Nicotinamide adenine dinucleotide (NAD) and its reduced form, NADH, serve as essential cofactors in energy metabolism. Because of NAD’s inherent characteristics as a small, hydrophilic molecule bearing a negative charge, which restricts its membrane permeability, cellular uptake traditionally requires extracellular degradation into its precursor metabolites (nicotinamide and nicotinamide riboside) to allow intracellular transport for subsequent enhancement of NAD biosynthesis. This conversion mechanism exhibits limited efficiency due to regulatory constraints imposed by rate-limiting enzymes such as NAMPT. Ye et al. [86] engineered NAD(H)-loaded nanoparticles to enable direct cellular uptake of NAD(H). The NAD(H)-loaded NPs are fabricated by using a calcium phosphate (CaP) or metal-organic framework (MOF) nanoparticle to encapsulate NAD/NADH, followed by coating with a lipid bilayer. Within the NAD(H)-loaded NPs, the CaP or MOF core enables them to dissolve in the acidic endosomal environment, leading to endosomal swelling and rupture, which releases the encapsulated NAD(H) into the cytosol (Fig. 7B). Treatment with NAD(H)-NPs markedly reduced the cytosolic calcium concentrations and prevented the loss of mitochondrial membrane potential, thereby enhancing cellular energy supply, averting pyroptosis and apoptosis, and ultimately attenuating inflammatory responses to suppress the progression of sepsis. This study demonstrates that organic nanomaterials can precisely modulate macrophage function via targeted delivery of immunomodulatory molecules, effectively reversing their pro-inflammatory phenotype and reducing inflammatory cytokine levels.

#### Biomimetic membrane-coated biomaterial-mediated macrophage modulation strategies

Biomimetic cell membrane-coated nanoparticles preserve the membrane proteins and recognition functions of the source cells, enabling prolonged circulation and active targeting—particularly for regulating immune cells such as macrophages [87]. In these biomimetic nanoparticles, membranes derived from key immune components, including macrophages, neutrophils, and mesenchymal stem cells, cancer cells, or even bacterial sources, are utilized as coating materials, preserving their native structural and functional characteristics. This preservation allows the nanoparticles to evade immune surveillance by the host’s immune system [88]. Such immune-evasion capability allows the nanoparticles to maintain a prolonged presence in the bloodstream and be effectively utilized at inflammatory sites [89]. Unlike artificial liposomes that merely mimic cell membranes, these cell membrane-coated nanoparticles can precisely replicate the intricate biomolecular milieu of natural cell surfaces. This is accomplished by maintaining the native composition and functionality of the source cell membranes, encompassing lipids, glycans, and proteins [90,91]. For instance, macrophage membranes retain the ability of their parent cells to bind endotoxins and pro-inflammatory cytokines; coating nanoparticles with such membranes has been shown to reduce pro-inflammatory cytokine levels and suppress bacterial dissemination in septic mice [92].

Beyond passive neutralization, these platforms can be engineered for active therapeutic delivery. A pivotal concern in sepsis management is the excessive production of ROS, which induces oxidative stress and may culminate in multiorgan failure. Qu et al. [93] engineered an antioxidative nanoplatform (designated mAOI NP) for enhancing the delivery and efficacy of antioxidants. The mAOI NP is fabricated by coordinating the antioxidant TA with ferric iron (Fe^3+^) to form TA-Fe nano-complexes, followed by the introduction of the flavonoid quercetin (Qu) to terminate excessive polymerization and control the self-assembly process; finally, the composites are coated with macrophage membranes. Within the mAOI NP, the macrophage membrane enables homologous targeting to inflammatory sites, while the TA/Qu core effectively scavenges ROS, repairs mitochondrial damage, and up-regulates the Nrf2/HO-1 antioxidant pathway. Furthermore, internalized mAOI NP promotes the polarization of pro-inflammatory M1 macrophages toward the anti-inflammatory M2 phenotype, demonstrating a combined antioxidative and immunomodulatory effects.

**Fig. 8.**
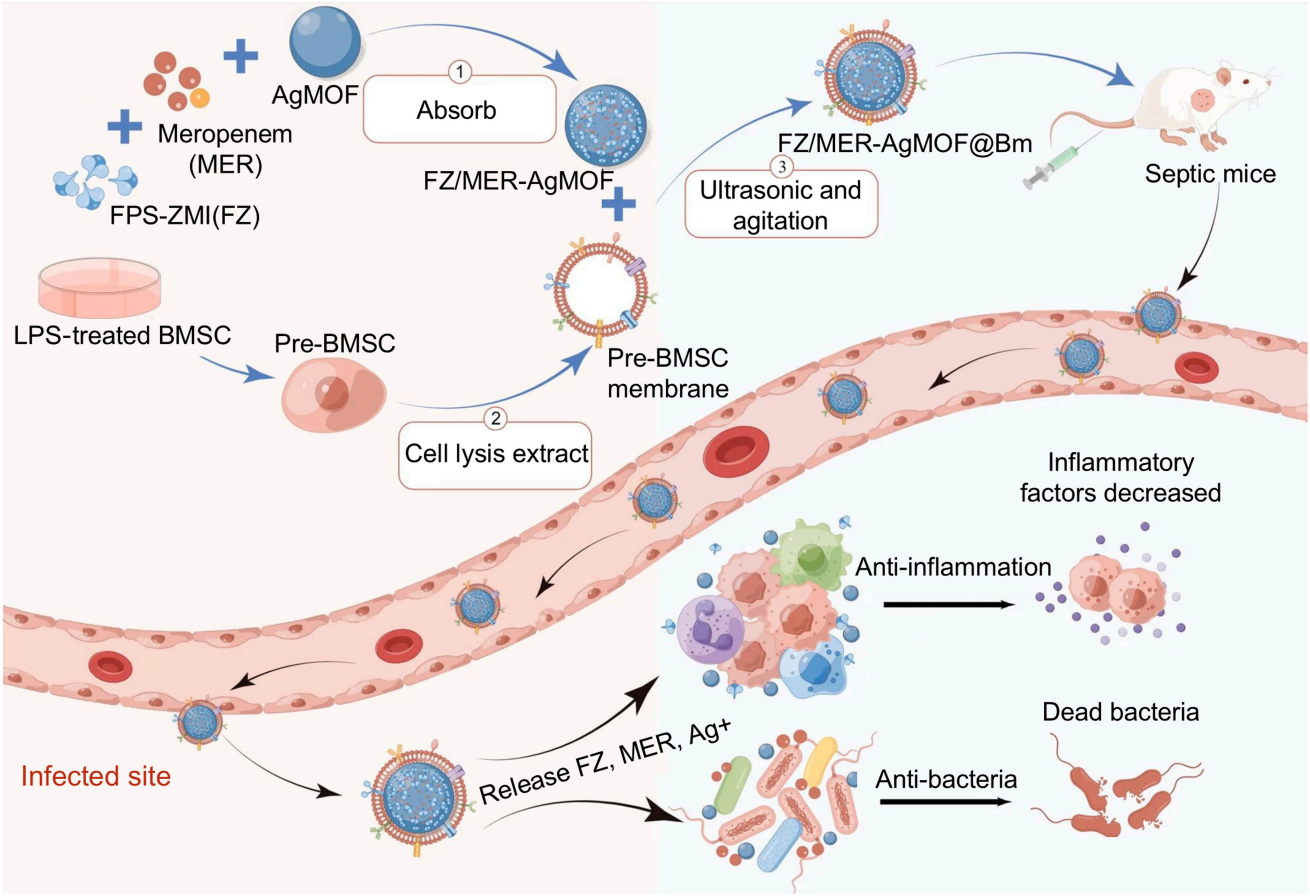
Schematic illustration of FZ/MER-AgMOF@Bm nanoparticles for sepsis treatment. Within the infection site, these nanoparticles combined with BMSC membrane coating for immune evasion and targeted delivery, along with an AgMOF core coencapsulating the anti-inflammatory agent FPS-ZM1 and the antibiotic meropenem, enable synergistic antimicrobial and immunomodulatory effects [95]. Copyright 2023, *J Nanobiotechnology*.

Stem cell membranes offer another promising source for biomimetic coatings, given their inherent immunomodulatory properties. Bone marrow mesenchymal stromal cells (BMSCs) are known to alleviate sepsis pathophysiology by modulating immune dysregulation and coagulation [94]. For instance, BMSCs express abundant immunosuppressive ligands such as programmed cell death-1 (PD-1) and Fas ligand, which minimize monocyte infiltration and limit the release of pro-inflammatory cytokines, while also exhibiting high expression of ICAM-1 and VCAM-1 to target inflammatory microenvironments. Lu et al. [95] engineered a multifunctional nanoparticle (designated FZ/MER-AgMOF@Bm) for sepsis management. The FZ/MER-AgMOF@Bm is fabricated by a silver metal-organic framework (AgMOF) as the core carrier, loaded with the receptor for advanced glycation end-products (RAGE) inhibitor FPS-ZM1 and the antibiotic meropenem (MER), and modified with LPS-primed BMSC membranes. Within the FZ/MER-AgMOF@Bm, the BMSC membranes facilitate targeted delivery to infection sites and promote macrophage M2 polarization, while the core components synergistically inhibit the HMGB1/RAGE axis and exert antibacterial effects (Fig. 8). Thus, by leveraging the natural targeting and signaling functions of source cell membranes, the biomimetic membrane-coated biomaterials achieved precise localization to inflammatory sites and enabled multifaceted modulation of macrophage activity, thereby offering a potent and sophisticated tool for sepsis immunomodulation.

#### EV biomaterial-based macrophage modulation strategies

**Fig. 9.**
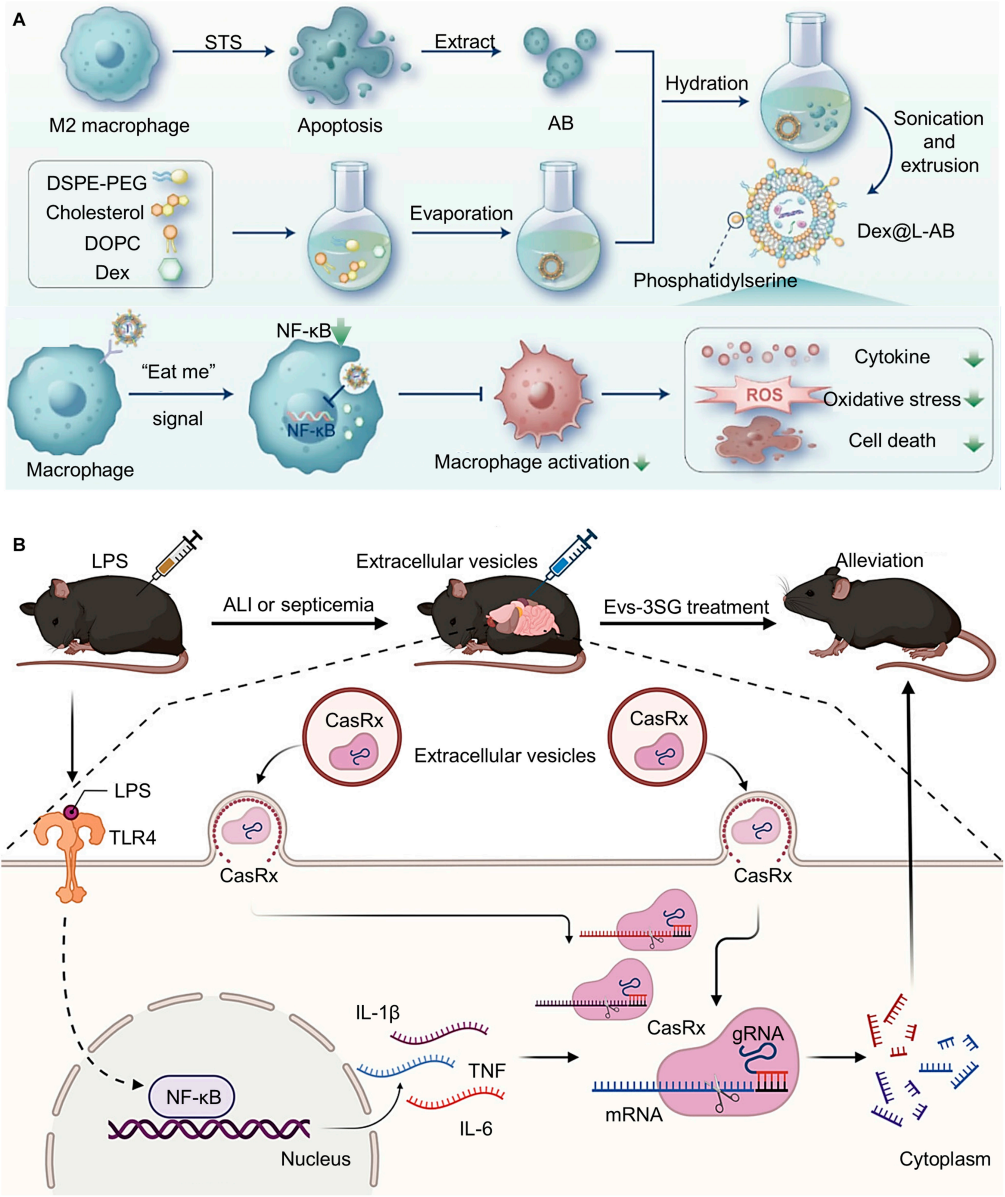
Extracellular vesicles biomaterial-based macrophage modulation strategies for sepsis treatment. (A) Schematic illustration of apoptotic body-based biomimetic hybrid nanovesicles for sepsis therapy. The nanovesicles are engineered to be recognized and internalized by macrophages via “eat me” signals, subsequently releasing the loaded dexamethasone to suppress the NF-κB pathway and inhibit macrophage activation in sepsis. Reprinted with permission from Ref. [100]. Copyright 2024, *J Nanobiotechnology*. (B) Schematic illustration of engineered extracellular vesicles for delivering the CRISPR/CasRx RNA-editing system to macrophages. These vesicles facilitate transient knockdown of key pro-inflammatory cytokine RNAs (IL-6, TNF, and IL-1β), thereby modulating inflammatory responses in sepsis. Reprinted with permission from Ref. [106]. Copyright 2023, *Sci. Adv*.

EVs are garnering growing interest as anti-inflammatory therapeutics and drug delivery platforms, owing to their inherent biocompatibility, low immunogenicity, and natural targeting capabilities [96]. These nano-sized membrane-bound vesicles, secreted by diverse cell types, include apoptotic bodies (ABs), microvesicles, and exosomes. As endogenous nanoparticles, EVs exhibit significant potential for sepsis treatment owing to their ability to facilitate intercellular communication through the transfer of bioactive molecules, including proteins, nucleic acids, and lipids [97]. Several nanomedicines employing EVs as delivery vehicles have been developed to modulate inflammatory responses. To optimize drug loading, various techniques have been devised for incorporating therapeutics into EVs, such as ABs, encompassing both passive and active loading methods [98]. However, these approaches are often constrained by complexity, suboptimal efficiency, and potential loss of bioactivity. Prior research has demonstrated that hybrid nanovesicles, generated through the fusion of liposomes with natural nanovesicles, exhibit high drug-encapsulation efficiency, preserve biological functionality, and enhance targeting capacity [99]. For instance, given the anti-inflammatory properties of M2 macrophages and their derived ABs, Lan et al. [100] engineered an AB-based biomimetic hybrid nanovesicle (L-AB) for utilizing the specific uptake of macrophages. The L-AB is fabricated by combining ABs with artificial liposomes to encapsulate dexamethasone (Dex). This design allows L-AB to inherit “eat me” signaling molecules present on the AB surface, thereby facilitating targeted uptake by macrophages. This design enabled efficient intracellular delivery of Dex, which suppressed macrophage hyperactivation and oxidative stress by inhibiting the NF-κB pathway, ultimately improving survival in septic mice (Fig. 9A).

In addition to small-molecule delivery, EVs also offer a promising vehicle for nucleic acid-based therapeutics. The CRISPR/Cas system represents a powerful tool for precision gene regulation, but its clinical translation has been hampered by the lack of safe and efficient delivery vectors. While viral vectors currently serve as the predominant delivery tools [101], their immunogenicity and potential for insertional mutagenesis limit clinical translation [102]. EVs offer a promising alternative due to their low immunogenicity and natural delivery capacity. Recent advances have demonstrated that EVs can efficiently deliver CRISPR-Cas9/guide RNA (gRNA) complexes for genome editing [103]. Compared to the CRISPR/Cas9 system, which induces permanent genomic modifications, CRISPR/Cas13d (CasRx) represents a relatively safer alternative as it directly recognizes and cleaves target RNA, enabling transient transcriptome modulation [104,105]. Li et al. engineered an EV-based nanoparticle (designated CasRx–gRNA EVs) for safe and efficient modulation of inflammation at the RNA level. The CasRx–gRNA EVs are fabricated by EVs derived from HEK293T cells, loaded with the CRISPR/CasRx system and tandem gRNAs targeting key cytokines (IL-6, TNF-α, and IL-1β) [106]. Upon delivery via EVs, the CasRx–gRNA complex transiently disrupted the RNA of the target cytokines, thereby suppressing their activation, reducing systemic inflammation, and improving outcomes in septic models (Fig. 9B). These studies highlight the versatility of EV-based platforms in delivering diverse therapeutic cargoes to precisely modulate macrophage function in sepsis.

#### Inorganic biomaterial-based macrophage modulation strategies

**Fig. 10.**
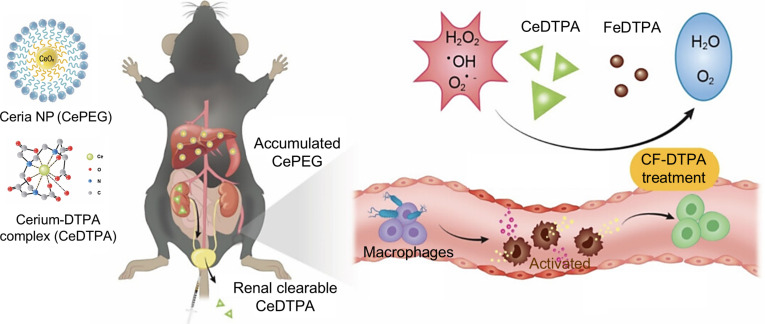
Schematic illustration of renal-clearable ceria-based nanoparticles (CeDTPA) in combination with Fe-DTPA (CF-DTPA) for sepsis treatment. These CF-DTPA nanoparticles synergistically scavenge hydrogen peroxide through the Fenton reaction, thereby alleviating macrophage activation and systemic inflammation. Reprinted with permission from Ref. [107]. Copyright 2024, *ACS Nano*.

Inorganic nanomaterials possess distinct advantages for sepsis treatment, including potent antioxidant and anti-inflammatory activities. However, their clinical translation has been limited by concerns regarding prolonged in vivo retention, potential systemic toxicity, and accumulation in the mononuclear phagocyte system. Through rational material design and metabolic engineering, these limitations can be mitigated. For instance, cerium-based nanomaterials typically form insoluble precipitates with physiological phosphates, leading to organ accumulation. However, complexing cerium with diethylenetriamine pentaacetic acid (DTPA) generates renal-clearable nanoparticles, preventing cerium ion leaching and systemic toxicity. Based on this principle, Kim et al. [107] constructed renal-clearable cerium-based nanoparticles derived from cerium–DTPA complexes. Ce-DTPA, in combination with Fe-DTPA (CF-DTPA), acts as an antioxidant pair that synergistically scavenges hydrogen peroxide via a Fenton-like reaction. The CF-DTPA reduced pro-inflammatory gene expression in LPS-activated macrophages and improved survival in septic mice by alleviating systemic inflammation and downstream tissue damage in the liver, spleen, and kidneys (Fig. 10). Therefore, metabolically optimized inorganic nanomaterials like CF-DTPA can serve as nonaccumulative nanomedicines, overcoming the limitations of conventional inorganic nanoparticle therapies.

Furthermore, carbon dots (CDs) have attracted growing interest in biomedicine owing to their exceptional biocompatibility, low cytotoxicity, and notable antibacterial properties. By exploiting the carbonizable components of bacterial cell walls, Li et al. [108] synthesized CDs from *Escherichia coli* (E-CDs) via pyrolysis carbonization. They demonstrated that E-CDs competitively inhibited LPS binding by targeting LPS-binding protein (LBP) and promoted LPS degradation via the lysosomal pathway. This interaction reduced excessive activation of the TLR4/NF-κB pathway in macrophages while effectively suppressing oxidative stress and overactivation of the STING pathway, thereby inhibiting the cytokine storm in sepsis. This strategy not only highlights a sustainable approach to converting pathogens into therapeutic agents but also exemplifies how inorganic nanomaterials can be designed to safely target key inflammatory pathways in macrophages.

### Nanomedicine for neutrophil immunomodulation

**Fig. 11.**
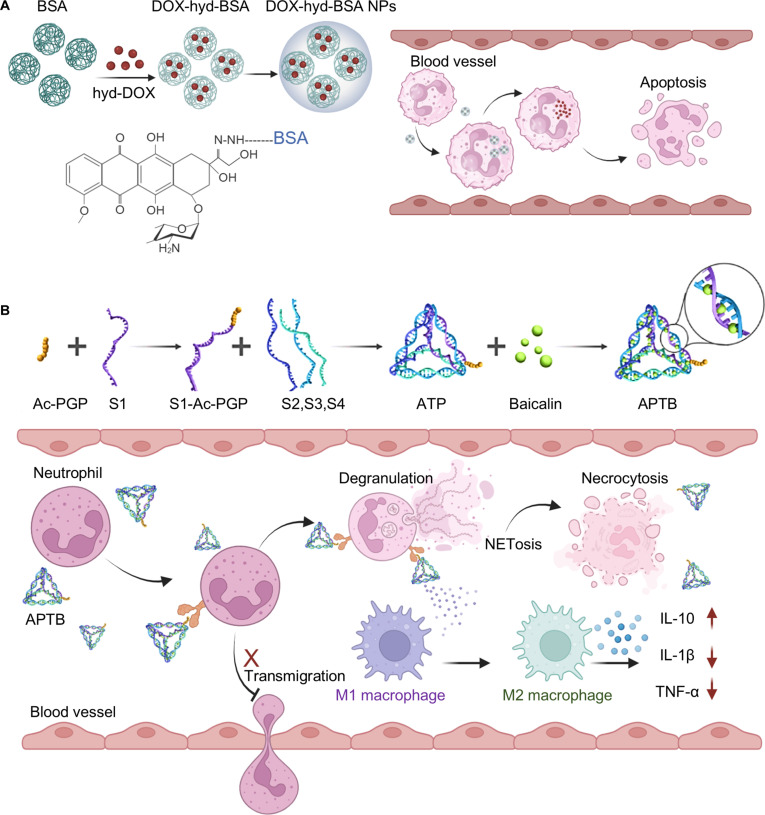
Organic biomaterial-based neutrophil modulation strategies for sepsis treatment. (A) Schematic illustration of DOX conjugated to BSA via a hydrazone bond to form DOX-hyd-BSA nanoparticles for sepsis treatment. Under the low-pH environment within neutrophils, DOX is released from the nanoparticles, thereby inducing neutrophil apoptosis. Reprinted with permission from Ref. [111]. Copyright 2019, *Sci. Adv*. (B) Schematic illustration of a targeted nanodrug delivery platform (Ac-PGP-tFNA, APT) that “hitchhikes” on circulating neutrophils by targeting CXCR2 to deliver baicalin. This platform delivers baicalin to promote neutrophil apoptosis and modulate macrophage polarization for sepsis treatment. Reprinted with permission from Ref. [114]. Copyright 2025, *ACS Nano*.

In severe sepsis, pathological stimuli disrupt the normal apoptotic pathway in neutrophils, steering them toward alternative cell-death modalities that amplify inflammation and contribute to organ dysfunction [109]. Nanoplatforms have been engineered to specifically target these hyperactive neutrophils, induce programmed cell death, and mitigate sepsis severity. To deliver doxorubicin (DOX) specifically into activated neutrophils, Wang et al. [110] engineered a sialic acid (SA)-functionalized nanoparticle loaded with DOX (DOX-SAL). Within the DOX-SAL, the SA moiety binds to L-selectin (CD62L) on circulating inflammatory neutrophils, thereby enabling targeted DOX delivery and subsequent induction of apoptosis, and suppressing inflammation. In another study, Zhang et al. synthesized a pH-sensitive DOX delivery system (DOX-hyd-BSA) for inducing programmed cell death and inhibiting neutrophil migration. The DOX-hyd-BSA is fabricated by conjugating DOX to the bovine serum albumin (BSA) through a hydrazone bond (hyd) [111], which degrades in the acidic microenvironment of activated neutrophils, leading to the release of DOX from the BSA conjugate (Fig. 11A). In septic mice, these nanoparticle strategies significantly reduced cytokine levels and suppressed systemic inflammatory by promoting apoptosis of inflammatory neutrophils, thereby markedly improving survival.

Tetrahedral framework nucleic acids (tFNAs) are recognized as one of the most versatile framework DNA nanomaterials, widely utilized in immunomodulation and drug delivery due to their superior cellular uptake, tissue permeability, structural editability, and biocompatibility [112]. These tFNAs can be functionalized with drugs or aptamers for enhanced targeting and therapy [113]. By leveraging a tFNA structure combined with a neutrophil-hitchhiking mechanism, Zhou et al. [114] engineered a targeted nanodrug delivery platform termed Ac-PGP-tFNA (APT) for delivering the anti-inflammatory agent baicalin (designated APTB). Within the APTB, the tFNA framework is functionalized with the N-acetyl-Pro-Gly-Pro (Ac-PGP) peptide, which specifically targets neutrophils by binding to the C-X-C motif chemokine receptor 2 (CXCR2) on the neutrophil membrane, enabling a “hitchhiking” delivery mechanism. APTB significantly enhanced baicalin’s bioavailability, promoted neutrophil apoptosis, reduced NETs formation, and modulated macrophage polarization toward the M2 phenotype (Fig. 11B). This study underscores the potential of functionalized nanomaterials for the targeted regulation of activated neutrophils, thereby systemically mitigating sepsis-associated inflammation and tissue injury.

### Nanomedicine for reversing immunoparalysis

#### Organic biomaterial-based immunosuppression reversal strategies

**Fig. 12.**
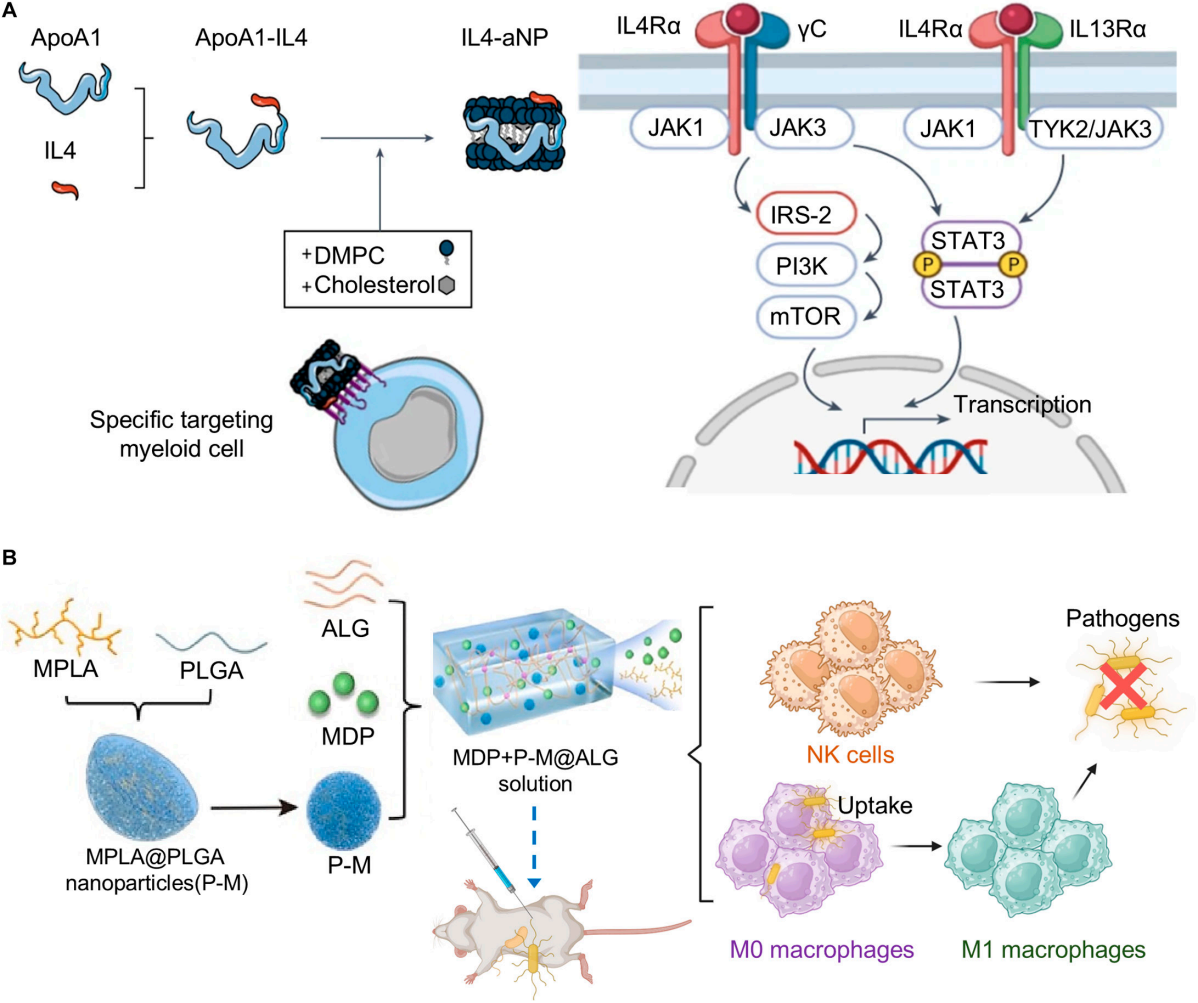
Organic biomaterial-based immunosuppression reversal strategies for sepsis treatment. (A) Schematic diagram of the IL4-aNP for sepsis treatment, which is fabricated by encapsulating a fusion protein consisting of apoA1 and IL4 (apoA1-IL4) within lipid nanoparticles. The nanoparticles target myeloid cells to deliver IL4, stimulate the key signaling pathway STAT6 to suppress acute inflammation, and PI3K-mTOR to induce trained immunity to reverse immunoparalysis. Reprinted with permission from Ref. [117]. Copyright 2023, *Nat. Biomed. Eng*. (B) Schematic illustration of a 2-phase releasing immunostimulatory composite (MDP + P-M@ALG) for sepsis treatment. This composite is fabricated by encapsulating the TLR4 agonist monophosphoryl lipid A (MPLA) into PLGA nanoparticles (P-M), and then combining with the NOD2 agonist muramyl dipeptide (MDP) and alginate (ALG), which enables fast and sustained release of immune-stimulating agents, providing effective and long-term protection against sepsis. Reprinted with permission from Ref. [120]. Copyright 2021, *Biomaterials*.

In the late stage of sepsis, immunoparalysis increases the risk of secondary infections. Conventional immune stimulants often exhibit transient efficacy and lack specificity. Nanocarriers enable sustained release of immunomodulators, which can induce innate immune memory and restore immune cell function to provide long-term immunoprotection. IL4 is known for its anti-inflammatory properties [115,116], but its clinical translation is impeded by poor pharmacokinetics. To address this, Schrijver et al. [117] engineered IL4-containing nanoparticles (IL4-aNPs) for effectively exerting immunomodulatory function in inflammatory conditions. The IL4-aNPs are fabricated by encapsulating a fusion protein consisting of apoA1 and IL4 (apoA1-IL4) within lipid nanoparticles. Within the IL4-aNPs, apoA1 facilitates targeted delivery to myeloid cells, while IL4 exerts acute anti-inflammatory effects via the STAT6 pathway and concurrently induces persistent innate immune memory (“trained immunity”) through the PI3K-mTOR pathway. Treatment with IL4-aNPs reduced TNF-α and IL-6 production in LPS-stimulated monocytes and enhanced long-term monocyte reactivity, successfully reversing sepsis-induced immunoparalysis in models (Fig. 12A).

Many sepsis survivors experience prolonged immunosuppression, characterized by immune cell dysfunction and increased susceptibility to secondary infections [28]. Activation of NLR and TLR signaling triggers cytokine release that supports immune cell function [118,119]. Based on this, Zhao et al. [120] engineered immunostimulatory composite nanoparticles with biphasic release characteristics (designated MDP + P-M@ALG) to enhance anti-infective activity and eliminate invading pathogens. The composite nanoparticles are fabricated by encapsulating the TLR4 agonist monophosphoryl lipid A (MPLA) into PLGA nanoparticles (P-M), then combining with the NOD2 agonist muramyl dipeptide (MDP) and alginate (ALG) (Fig. 12B). Within MDP + P-M@ALG, the small-molecule MDP exhibited rapid release upon injection and provided transient protection against *E. coli* infection by activating innate immune cells, while MPLA within the P-M nanoparticle exhibits sustained release, enabling long-term immune modulation and conferring broad protection against various pathogens. Treatment with MDP + P-M@ALG increased bacterial uptake; elevated proportions of M1 macrophages, neutrophils, and NK cells; and induced stronger cytokine secretion in immunosuppressed mice, thereby improving survival and providing long-term protection against secondary infections. These studies demonstrate that nanocarrier-delivered immunomodulators not only exert acute anti-inflammatory effects but also enhance long-term anti-infection capacity through trained immunity, offering a temporal immunorestoration strategy for sepsis during the immunoparalytic phase.

#### Cell therapy-based immunosuppression reversal strategies

**Fig. 13.**
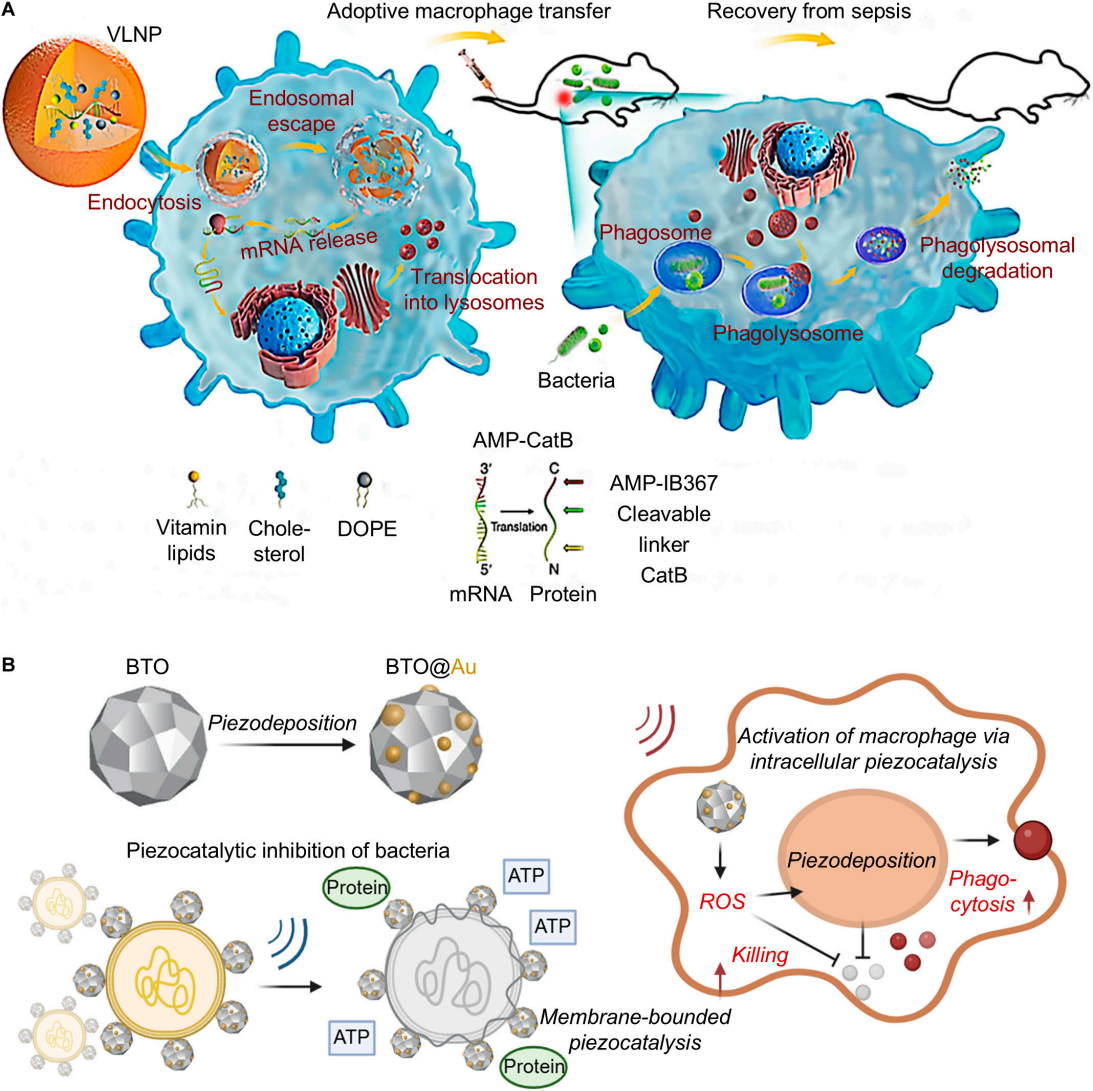
Cell therapy-based immunosuppression reversal strategies for sepsis treatment. (A) Schematic illustration of V_C_LNP for delivering AMP-CatB mRNA to macrophages in sepsis. Upon lysosomal entry, the nanoparticles release AMP-IB367 to eradicate ingested multidrug-resistant bacteria. Reprinted with permission from Ref. [125]. Copyright 2020, *Nat. Nanotechnol*. (B) Schematic illustration of piezoelectric nanoparticles (BTO@Au NPs) for enhancing macrophage function in sepsis. The piezo-nanoparticles are fabricated by piezodeposition of Au on BaTiO_3_ (BTO) nanoparticles. Under ultrasound activation, intracellular piezocatalysis promotes macrophage phagocytosis and killing of bacteria. Reprinted with permission from Ref. [127]. Copyright 2025, *Nat. Commun*.

Numerous bacteria evolve immune-evasion strategies that thwart phagocytosis and lysosomal killing, leading to persistent and recurrent infections [121,122]. The direct application of immunostimulants often fails to restore the function of damaged macrophages and monocytes [123]. In immunosuppressed sepsis patients, nanoplatforms can potentiate immune responses by delivering immunostimulants or antigens to target immune cells and bolster microbial defense [124]. Hou et al. [125] engineered an mRNA delivery platform (designated AMP-cat-B@VLNP) for efficient intracellular delivery of mRNA encoding bactericidal AMPs. The AMP-cat-B@VLNP is fabricated by encapsulating mRNAs encoding the AMP AMP-IB367 and cathepsin B (CatB) within vitamin C-derived lipid nanoparticles. Within the AMP-cat-B@VLNP, vitamin C promotes the specific accumulation of the nanoparticles in macrophage lysosomes, which serve as the crucial site for bactericidal activities. Upon acquiring AMP-cat-B@VLNP, macrophages translate the mRNA into functional cytosolic proteins. These proteins subsequently translocate to lysosomes, where the linker is cleaved by CatB, thereby releasing the active AMP AMP-IB367. When bacteria-containing phagosomes fuse with these lysosomes, internalized bacteria are exposed to both pre-released AMP-IB367 and innate lysosomal antimicrobial components, effectively eliminating MDR pathogens that evade conventional killing (Fig. 13A). Adoptive transfer of macrophages loaded with AMP-cat-B@VLNP cleared MDR bacteria in immunosuppressed septic mice and prolonged survival.

Persistent infections caused by methicillin-resistant *Staphylococcus aureus* (MRSA) and the subsequent induction of host immunoparalysis stand as major factors contributing to sepsis-related mortality. Existing clinical interventions often fail to fully restore immune homeostasis and eliminate MRSA. To stimulate innate immunity against MRSA, Tang et al. [126] engineered macrophage-targeting peptide CRV (sequence CRVLRSGSC)-modified lipid nanoparticles (CRV/LNP-RNAs) for the transient in situ programming of macrophages (MΦs). The CRV/LNP-RNAs are fabricated by coencapsulating mRNA encoding an MRSA-targeted chimeric antigen receptor (SasA-CAR mRNA) and CASP11 siRNA (a key MRSA intracellular evasion target), together with the CRV peptide. Within the CRV/LNP-RNAs, the CRV peptide binds to the RXRβ receptor, enabling targeted RNA delivery to macrophages at both tumor and infection sites, thereby generating CAR-MΦs with enhanced bactericidal potency. This strategy demonstrated that the engineered MΦs could efficiently phagocytose and intracellularly digest MRSA, thereby preventing immune evasion by this “superbug”.

Capitalizing on catalytic antibacterial nanoparticles that generate ROS, Liu et al. [127] engineered a piezocatalytic nanoparticle (designated BaTiO₃@Au) as an ultrasound-responsive nanocatalyst for stimulating macrophage immune responses. The BaTiO_3_@Au is fabricated by pieodeposition of Au on BaTiO_3_ (BTO) nanoparticles. Under ultrasound exposure, these piezoelectric nanoparticles (piezoNPs) significantly enhanced ROS generation within macrophages, thereby boosting their antibacterial phagocytosis and killing activity (Fig. 13B). Mouse bone marrow-derived macrophages were incubated with piezoelectric nanoparticles to form piezoelectric macrophages (piezo-Mφ). Adoptive transfer of piezo-Mφ in a chronic infection model exhibited long-term antibacterial efficacy, highlighting the potential of catalytic nanoparticles to modulate innate immune responses and engineer macrophages for immunotherapy. This highlights that nano-engineered cellular therapies establish a novel living-cell therapeutic paradigm, where modified immune cells exhibit enhanced bactericidal and immunomodulatory capacities to combat drug-resistant infections and reverse immunoparalysis in sepsis.

## Summary of Advantages and Limitations of Biomaterials for Sepsis

Despite the considerable preclinical potential of nanomedicine in sepsis management, most therapeutic strategies remain limited to preliminary research phases, with their clinical translation requiring resolution of multiple significant barriers. These include not only fundamental issues of material safety, biodistribution, metabolism, and long-term toxicity, but also practical hurdles in scalability, reproducibility, batch-to-batch consistency, and regulatory approval. The distinct classes of nanomaterials—organic, inorganic, and biomimetic—each face unique obstacles in the path toward clinical application.

Organic biomaterials (e.g., PLGA nanoparticles, liposomes, and polymeric micelles) are generally biodegradable and exhibit high biocompatibility. They are capable of encapsulating a diverse range of therapeutic agents—such as antibiotics, anti-inflammatory drugs, nucleic acids, and antioxidants—and can be engineered for controlled drug release, offering significant advantages in sepsis therapy [128]. Furthermore, several organic nanocarriers, particularly liposomes and PLGA-based nanoparticles, have received Food and Drug Administration (FDA) approval for use in other disease areas. This provides relatively established regulatory pathways that could potentially accelerate their clinical translation for sepsis [129]. However, the clinical translation of organic biomaterials for sepsis still faces a series of challenges. Rapid recognition and clearance by the mononuclear phagocyte system (MPS) remain a major obstacle [130]. Despite the fact that surface modification with PEG or other “stealth” coatings is commonly employed to prolong circulation time, many organic nanoparticles are still rapidly opsonized and recognized by complement proteins and macrophages [131]. Concurrently, PEG modification may trigger the production of anti-PEG antibodies, leading to the accelerated blood clearance phenomenon and complement activation-related pseudoallergy, a hypersensitivity syndrome that poses significant clinical safety concerns [132]. Furthermore, while organic materials are readily engineered as microenvironment-responsive systems, they also face challenges related to uncontrolled drug release. For instance, Zhao et al. engineered immunostimulatory composite nanoparticles with biphasic release characteristics (designated MDP + P-M@ALG) to enhance anti-infective activity and eliminate invading pathogens. Within the MDP + P-M@ALG, the TLR agonist intended for slow, sustained release during the immunosuppressive phase of sepsis may undergo premature release or leakage due to the complex pathological microenvironment. This could potentially induce a hyperinflammatory state, thereby exacerbating septic injury [120].

Inorganic nanomaterials (e.g., gold, silver, and manganese-based nanoparticles) provide intrinsic antioxidant properties that can be harnessed for sepsis therapy. Yet, their clinical translation is severely constrained by concerns over long-term tissue retention and potential toxicity. For instance, manganese-based systems like TMP, while effective at scavenging ROS and cfDNA, risk accumulation in the basal ganglia, potentially inducing neurotoxicity reminiscent of Parkinsonian syndromes [75]. Cerium-based nanoparticles, despite potent antioxidative activity, tend to precipitate with physiological phosphates and are avidly taken up by the reticuloendothelial system, leading to prolonged organ retention. Although strategies such as chelation engineering (e.g., DTPA conjugation) or the design of ultrasmall, renal-clearable particles have shown promise in mitigating these effects [107], their long-term safety profiles in humans remain largely unexplored.

Biomimetic platforms, particularly cell membrane-coated nanoparticles and EVs, offer unparalleled advantages in immune evasion, homologous targeting, and the ability to neutralize a broad spectrum of inflammatory mediators. However, they face some of the most daunting translational challenges. The production of cell membrane-derived coatings or EVs at a clinical scale is inherently complex and suffers from poor batch-to-batch reproducibility, as membrane composition can vary with cell culture conditions, passage number, and isolation methods [96,133,134]. Efficient and consistent loading of therapeutic cargoes into these vesicles remains technically challenging, often resulting in low encapsulation efficiencies and heterogeneous formulations. Furthermore, the regulatory classification of such bio-derived products is ambiguous—should they be regulated as drugs, biologics, or combination products? Rigorous characterization of their immunogenicity and off-target effects is required before clinical deployment [135].

Beyond material-specific hurdles, several cross-cutting issues impede clinical translation. The in vivo fate of nanomedicines—including their biodistribution, cellular uptake, metabolism, and clearance pathways—is incompletely understood in the context of sepsis, where organ dysfunction and altered hemodynamics can dramatically shift these parameters. Long-term toxicity data are scarce, particularly for novel materials or repeated dosing regimens. Scalability and manufacturing reproducibility remain major bottlenecks, especially for complex, multicomponent nanoparticles. Finally, the regulatory landscape for nanomedicines in sepsis is still evolving: there are no specific FDA or European Medicines Agency guidelines tailored to sepsis nanotherapeutics, and the lack of standardized characterization methods complicates the comparison and evaluation of different platforms [135]. Collectively, these challenges underscore that the successful translation of sepsis nanomedicines requires a holistic, mechanistic understanding of each material’s unique characteristics and limitations—only by thoroughly elucidating their behavior in the septic milieu, establishing robust safety profiles, and ensuring reproducible manufacturing can we harness their therapeutic advantages while mitigating off-target effects and toxicity. These underscore that the development of sepsis nanomedicines requires a holistic approach that integrates material design with rigorous assessment of manufacturability, safety, and regulatory compliance.

Encouragingly, recent efforts toward clinical translation are actively underway. A recent Phase IIa clinical trial has evaluated the therapeutic application of phospholipid nanoparticles in septic shock management. This investigational approach employs a phospholipid nanoparticle platform to develop an intravenous infusion system based on “VBI-S phospholipid nanoparticles”. The nanoparticles demonstrate reversible adsorption capacity for scavenging excessive NO generated during septic episodes, which consequently provides multiorgan protective benefits, restores vascular reactivity to vasoactive agents, and enhances microcirculatory perfusion and oxygen delivery efficiency. In patients with refractory hypotension unresponsive to conventional fluid resuscitation, VBI-S infusion resulted in a mean arterial pressure (MAP) increase of ≥10 mmHg in 100% of patients. Within 48 h, statistically significant improvements were observed in oxygenation index, renal function, inflammatory markers, lactate levels, coagulation parameters (PT/INR), and Sequential Organ Failure Assessment scores, with no serious adverse events reported. Furthermore, a clinical study is ongoing to evaluate the synergistic immunomodulatory effects of nanoselenium capsules combined with alanyl-glutamine for sepsis treatment [136]. In this approach, nanoselenium leverages its antioxidant properties to scavenge excessively produced ROS during sepsis, thereby alleviating oxidative stress damage and suppressing hyperinflammation. Concurrently, glutamine provides energy support for proliferating and activated immune cells, promoting lymphocyte proliferation and functional recovery. This strategy aims to address the limitations of conventional sepsis therapies, which often struggle to effectively reverse immune dysfunction through anti-infective and organ support measures alone. These examples, while limited, signal a gradual pivot toward clinically translatable nanomedicine designs.

To summarize the main categories of biomaterial-based nanomedicine strategies, we provide an overview of each material type (organic, inorganic, and natural) and systematically describe their therapeutic mechanisms, targeting efficiency, immunomodulatory capacity, biodegradability, clearance rate and potential toxicity, as well as key advantages and limitations (Table 2).

**Table 2.** Summary of biomaterial-based nanomedicine strategies for sepsis treatment

Material category	Representative systems	Therapeutic mechanisms	Key advantages	Key limitations	Ref.
Organic biomaterials	Antimicrobial peptide nanofibers, PLGA nanoparticles, polymeric micelles, liposomes	Pathogen capture; ROS scavenging; inducing macrophage polarization (M1→M2); inducing neutrophil apoptosis	Ease to multitarget modification and stimuli-responsive design; high biocompatibility	Rapid clearance by the reticuloendothelial system; potential immunogenicity	[53–55], [78–81], [120–128]
Inorganic biomaterials	Gold nanoparticles, manganese-based nanoparticles (TMP), cerium-based nanoparticles, carbon dots	Bacterial disrupting; ROS/cfDNA/hydrogen peroxide scavenging; competitive inhibition LPS binding	High stability; high intrinsic antioxidant/catalytic properties	Poor biodegradability; long-term tissue retention concerns and RES uptake; neurotoxicity risk	[59,70], [74,75], [102,103]
Natural biomimetic materials	Cell membrane-coated NPs, extracellular vesicles (EVs), apoptotic bodies, cell therapy	ROS scavenging; inducing macrophage polarization (M1→M2); immunometabolic reprogramming of macrophage	High targeting capability and biocompatibility; low immunogenicity	Poor batch-to-batch reproducibility; low drug loading efficiency	[57,82], [91,92], [130–132]

PLGA, poly(lactic-co-glycolic acid); ROS, reactive oxygen species; TMP, nanoparticle containing tannic acid, manganese, and polymyxin B; LPS, lipopolysaccharide; NP, nanoparticle

## Exploratory Cases of Early Smart Nanomaterials

Biomaterials, with their programmable physicochemical properties, microenvironment responsiveness, and biocompatibility, offer a new avenue for the spatiotemporal management of sepsis. Early smart nanomaterials have been reported to exhibit preliminary “adaptive” or “programmable response” functionalities in the context of sepsis or related inflammatory conditions. Although these studies have not yet matured to clinical application, they have established foundational directions for research in smart materials. The complex pathological microenvironment of sepsis has inspired the design of various stimuli-responsive nanosystems, including pH-responsive-, ROS-responsive-, enzyme-responsive-, and pH/enzyme dual-responsive drug release systems or nanoparticles, which are designed to respond to specific pathological signals of sepsis.

For instance, Zhang et al. [60] developed a dual pH/enzyme-responsive polymeric micelle system for sepsis treatment. The polymer micelle, formed from a pH-sensitive copolymer serving as the backbone, enables the encapsulation of the antibiotic ciprofloxacin and the anti-inflammatory agent TPCA-1, while its surface is further functionalized with ICAM-1 antibodies to achieve targeted delivery. Within the polymer micelle, the pH-sensitive segments undergo conformational changes in response to the acidic microenvironment of the infection site, whereas enzyme-sensitive segments are cleaved by bacterial secretory enzymes. This dual mechanism synergistically triggers drug release at the infection site, thereby significantly reducing systemic drug exposure while mitigating adverse effects arising from nonspecific distribution of conventional antibiotics and systemic delivery of anti-inflammatory agents. Xiong et al. [137] constructed an enzyme-responsive nanogel drug delivery system for differential delivery of antimicrobials to bacterial infection sites. The system featured a polyphosphoester-crosslinked core encapsulating antibiotics, functionalized with an HA for targeted binding to the CD44 receptor expressed on macrophages. In this design, HA mediated the active targeting of the nanogels to macrophages at inflammatory sites. The polyphosphoester core was specifically degraded by enzymes secreted by bacteria (e.g., phosphatases) within the bacterial infection microenvironment, thereby triggering the precise release of the antibiotics. This strategy enabled targeted delivery and enzyme-controlled release of antibiotics specifically at bacterial infection sites, significantly enhancing the inhibition of bacterial growth. Wang et al. [138] developed ROS-responsive moxifloxacin-containing nanoparticles (MXF/Oxi-αCD NPs) for targeted delivery to infected lung tissue. This system was fabricated via a ROS-responsive material, i.e., 4-(hydroxymethyl) phenylboronic acid pinacol ester-modified α-cyclodextrin (Oxi-αCD), which was employed to encapsulate the antibiotic moxifloxacin. Upon reaching inflammatory sites, the locally high concentrations of ROS triggered the cleavage of the sensitive bonds, leading to the release of the antibiotic at the lesion site. This design achieved on-demand drug release specifically within the oxidative stress microenvironment, significantly reducing inflammation-induced pulmonary edema and inflammatory cell infiltration. Similarly, Ha et al. [80] developed a gold nanoparticle-photosensitizer hybrid system (AuNP-NSC) based on a photothermal response strategy. This system utilized AuNPs as the core, functionalized on the surface with NHCs and siderophores. Within the AuNP-NSC, the siderophores enabled specific targeting and entry into drug-resistant *P. aeruginosa* by binding ferric ions; the NHC acted as stabilizing ligands, exhibiting strong affinity for transition metals to form highly stable complexes with AuNPs via covalent bonding. Upon NIR light irradiation, the AuNPs generated localized hyperthermia and mechanical vibrations, synergistically disrupting the bacterial cell membrane and intracellular structures. Collectively, these strategies underscore a change from nonresponsive passive systems to smart stimuli-responsive platforms, enabling the achievement of spatiotemporally controlled release within the complex septic microenvironment.

However, current nanomedicine systems still face several limitations: (a) the responsiveness is typically restricted to 1 or 2 triggers, which proves insufficient for capturing the complex, evolving sepsis microenvironment; (b) drug release kinetics of nanomedicine may not align with the dynamic pathophysiological timeline; (c) achieving sequential or differential release of multiple cargoes remains challenging; and (d) long-term in vivo stability, off-target activation risks, and manufacturing scalability require further optimization. Nevertheless, these exploratory studies substantiate the fundamental principle that engineering materials capable of sensing and responding to the disease microenvironment provide an opportunity for designing more smart nanomaterial systems to adaptively respond to microenvironment changes in the future.

## Future Prospects and Challenges

This review systematically summarizes the recent advancements in biomaterial-based strategies for sepsis treatment, especially in immunomodulatory therapy. A diverse array of functional nanomaterials has demonstrated remarkable potential for restoring immune homeostasis and mitigating organ injury. These materials are commonly employed for sepsis treatment through multifaceted mechanisms, including pathogen and endotoxin scavenging, precise modulation of immune cell functions (e.g., macrophages and neutrophils), preservation of vascular endothelial integrity, and alleviation of immunoparalysis.

### Core scientific and engineering challenges in transitioning from single-function to dynamic adaptation

The tunable physicochemical properties, microenvironmental responsiveness, and inherent biocompatibility of nanomaterials offer promising strategies for modulating sepsis with temporospatial precision. However, despite significant advancements, most existing biomaterials primarily focus on a single pathological phase in sepsis therapy (e.g., targeting either pathogen clearance or immune modulation alone). The pathological microenvironment of sepsis exhibits high complexity, characterized by an initial cytokine storm followed by late-stage immunosuppression. Thus, these single treatment strategies remain a great challenge in improving therapeutic efficacy against sepsis due to their poor dynamic adaptability capacity. Future research should prioritize the development of advanced smart materials with real-time adaptive capabilities to respond to the dynamic microenvironmental changes across distinct pathological stages during sepsis progression.

While the promise of intelligent adaptive nanomaterials is evident, the transition from single-function systems to those capable of dynamic multistage adaptation presents substantial scientific and engineering challenges. First, the complexity of material design increases. The integration of multiple responsive elements, targeting ligands, and drugs into a single stable construct necessitates advanced synthetic and assembly techniques that frequently exhibit poor batch-to-batch reproducibility. Second, while our understanding of sepsis’s dynamic pathophysiology has advanced, significant knowledge gaps persist. Establishing precise thresholds for biomarkers (e.g., cytokine concentrations and redox balance) that distinguish between distinct pathological phases presents methodological challenges, which may result in suboptimal timing of therapeutic interventions. Third, maintaining an optimal equilibrium between safety and efficacy represents a critical challenge in clinical translation. Overly complex materials may provoke unintended immune responses or accumulate in off-target organs, whereas heightened responsiveness characteristics may precipitate undesired drug release kinetics or premature material degradation. These multifaceted challenges collectively emphasize the imperative for cross-disciplinary synergies in developing next-generation adaptive nanomaterials, with particular reliance on computational advancements provided by sophisticated artificial intelligence (AI) algorithms.

Recent advances in AI and machine learning (ML) have transitioned from conceptual speculation to concrete applications in nanomedicine design. For instance, researchers have developed a pattern-generating sensor array termed “NanoSA” by integrating a nanosensor array with ML algorithms for the rapid identification of sepsis-causing bacteria [139]. This system consists of a nanoassembly comprising 3 types of sensors, which generate 6-channel parallel sensing signals through intermolecular fluorescence resonance energy transfer (FRET) effects. After optimization using 9 ML algorithms, a multilayer perceptron (MLP) model was selected for pattern recognition and classification of the multidimensional spectral data generated by the sensors. This design achieved rapid identification of 24 sepsis-related bacterial species within just 30 s, with an accuracy of 96.9%. When tested on clinical sepsis samples, the MLP model demonstrated 97.6% accuracy in discriminating among 5 different bacterial infections. The sensor array also effectively distinguishes bacterial infection types and concentration gradients in different biological fluids, including serum and urine. This approach addresses the limitations of traditional blood culture methods, which are time-consuming (requiring 24 to 72 h) and prone to contamination, as well as the clinical challenge of identifying polymicrobial infections, thereby providing an efficient tool for rapid pathogen diagnosis in sepsis.

In another related study, a computational framework for optimizing the design of biomimetic nanosponges was developed by integrating computational pharmacology with ordinary differential equation (ODE)-based kinetic modeling [140]. This framework comprises an ODE kinetic model and a multiparameter optimization algorithm. An ODE kinetic model based on the law of mass action was first developed to simulate the competitive inhibition kinetics between biomimetic “nanosponges”—nanoparticles coated with erythrocyte membranes—and natural red blood cells for *S. aureus* α-toxin. Parameter sweeping was subsequently performed on nanoparticle radius and receptor surface density to identify the design window that maximizes toxin neutralization efficiency. This design enabled precise prediction and optimization of the kinetic characteristics of biomimetic nanosponges. Simulation results revealed that receptor surface density, rather than geometric surface area, is the primary determinant of “decoy” efficiency. The theoretical toxin neutralization efficiency within the optimal design window reached 91.79%. This work overcomes the limitations of traditional biomimetic nanoparticle development, which relies on empirical iteration and struggles to optimize competitive binding kinetics under high-flow hemodynamic conditions, thereby providing a computational framework for the rational design of next-generation nanotoxoid therapeutics.

### Future prospects

**Fig. 14.**
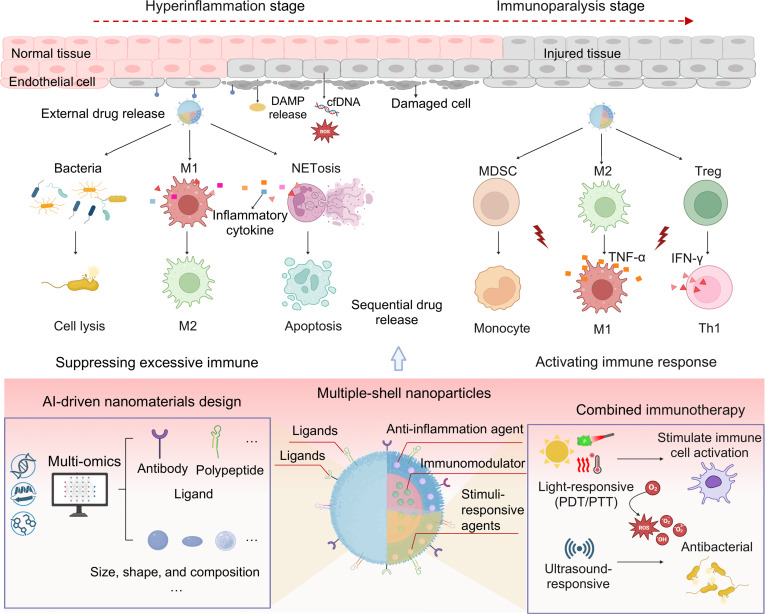
Schematic illustration of smart adaptive nanoparticles for sepsis treatment. The development of advanced smart materials with real-time adaptability to dynamic microenvironmental changes across distinct pathological stages during sepsis progression offers a novel therapeutic strategy for sepsis treatment. According to the complex pathological microenvironment of sepsis, advanced AI technologies can assist in high-throughput screening of optimal targeting and adaptive moieties, predicting biointerface interactions, and optimizing critical parameters, including size, surface chemistry, and release kinetics. The design and engineering of multiple-shelled, multistimuli-responsive nanoparticles may offer an alternative prospect. In these nanoparticles, the outer shells can be functionalized to encapsulate antimicrobial agents, while the inner core accommodates immunomodulators, thereby enabling responsive adaptation to dynamic microenvironmental changes across distinct pathological stages during sepsis progression. Furthermore, photothermal-regulated, photodynamic-regulated, or ultrasound-mediated immunoregulation may also synergistically recruit and enhance immune cell activity through coordinated mechanisms restoring their effector functions.

Future efforts may focus on designing intelligent, adaptive nanoparticles to achieve effective management across the full course of sepsis. Advanced AI technologies can be employed to facilitate high-throughput screening of optimal targeting and adaptability moieties, predict material–biointerface interactions, and optimize critical parameters such as size, surface chemistry, and release kinetics. This intelligent system is capable of dynamically adapting to the complex pathophysiology of sepsis. In the design and training of surface chemistries, the surface of the nanoparticles could be functionalized with multiple targeting ligands to respond to an excessive inflammatory and immunosuppressive microenvironment. In the design and training of nanoparticle topologies, training and developing multiple shells and multistimuli-responsive nanoparticles may provide an alternative prospect. Within these nanoparticles, the outer shells can be engineered to load antimicrobial agents, while the inner core permits loading of immunomodulators. This structural configuration enables autonomous adaptation to dynamic microenvironmental shifts through sequential drug release mechanisms. During the initial phase of cytokine storm development, when these nanoparticles respond to pathogens and inflammation, the exterior-loaded antimicrobial agents are preferentially released to suppress pathogen proliferation and mitigate inflammatory responses. Subsequently, during the late-stage immunosuppressive state, the nanoparticles respond to immunosuppressive cues by releasing their core-loaded immunomodulators, which act to reprogram immune cell function and restore effective immune surveillance. Moreover, these functional adaptive nanoparticles can be engineered as stimulus-responsive systems (e.g., photo-, ultrasound-, or ROS-sensitive materials), facilitating on-demand release of secondary therapeutics upon exposure to specific exogenous stimuli or endogenous signals (e.g., pH, enzymes, and ROS). In addition, photothermal, photodynamic, or ultrasound-mediated immunoregulation may be integrated to recruit and potentiate immune cells through synergistic mechanisms that restore their effector functions. These multifunctional intelligent platforms are anticipated to enable closed-loop management by dynamically responding to microenvironmental alterations across distinct pathological phases during sepsis progression, thereby substantially enhancing therapeutic outcomes and clinical prognosis in septic patients (Fig. 14).

## References

[1] Singer M, Deutschman CS, Seymour CW, Shankar-Hari M, Annane D, Bauer M, Bellomo R, Bernard GR, Chiche JD, Coopersmith CM, et al. The Third International Consensus Definitions for Sepsis and Septic Shock (Sepsis-3). JAMA. 2016;315(8):801–810. 26903338 10.1001/jama.2016.0287 PMC4968574

[2] Dellinger RP, Levy MM, Rhodes A, Annane D, Gerlach H, Opal SM, Sevransky JE, Sprung CL, Douglas IS, Jaeschke R, et al. Surviving sepsis campaign: International Guidelines for Management of Severe Sepsis and Septic Shock, 2012. Intensive Care Med. 2013;39(2):165–228. 23361625 10.1007/s00134-012-2769-8 PMC7095153

[3] Dellinger RP, Levy MM, Carlet JM, Bion J, Parker MM, Jaeschke R, Reinhart K, Angus DC, Brun-Buisson C, Beale R, et al. Surviving sepsis campaign: International Guidelines for Management of Severe Sepsis and Septic Shock: 2008. Crit Care Med. 2008;36(1):296–327. 18158437 10.1097/01.CCM.0000298158.12101.41

[4] Singer M. Antibiotics for sepsis: Does each hour really count, or is it incestuous amplification? Am J Respir Crit Care Med. 2017;196(7):800–802. 28504905 10.1164/rccm.201703-0621ED

[5] Raeven P, Zipperle J, Drechsler S. Extracellular vesicles as markers and mediators in sepsis. Theranostics. 2018;8(12):3348–3365. 29930734 10.7150/thno.23453 PMC6010985

[6] Makabenta JMV, Nabawy A, Li CH, Schmidt-Malan S, Patel R, Rotello VM. Nanomaterial-based therapeutics for antibiotic-resistant bacterial infections. Nat Rev Microbiol. 2021;19(1):23–36. 32814862 10.1038/s41579-020-0420-1 PMC8559572

[7] Hu Q, Sun W, Wang C, Gu Z. Recent advances of cocktail chemotherapy by combination drug delivery systems. Adv Drug Deliv Rev. 2016;98:19–34. 26546751 10.1016/j.addr.2015.10.022 PMC4998845

[8] Gafar MA, Omolo CA, Elhassan E, Ibrahim UH, Govender T. Applications of peptides in nanosystems for diagnosing and managing bacterial sepsis. J Biomed Sci. 2024;31(1):40. 38637839 10.1186/s12929-024-01029-2 PMC11027418

[9] Wang X, Xia Z, Wang H, Wang D, Sun T, Hossain E, Pang X, Liu Y. Cell-membrane-coated nanoparticles for the fight against pathogenic bacteria, toxins, and inflammatory cytokines associated with sepsis. Theranostics. 2023;13(10):3224–3244. 37351162 10.7150/thno.81520 PMC10283065

[10] Tu Z, Zhong Y, Hu H, Shao D, Haag R, Schirner M, Lee J, Sullenger B, Leong KW. Design of therapeutic biomaterials to control inflammation. Nat Rev Mater. 2022;7(7):557–574. 35251702 10.1038/s41578-022-00426-z PMC8884103

[11] Brannon ER, Guevara MV, Pacifici NJ, Lee JK, Lewis JS, Eniola-Adefeso O. Polymeric particle-based therapies for acute inflammatory diseases. Nat Rev Mater. 2022;7(10):796–813. 35874960 10.1038/s41578-022-00458-5 PMC9295115

[12] Ismail EA, Devnarain N, Govender T, Omolo CA. Stimuli-responsive and biomimetic delivery systems for sepsis and related complications. J Control Release. 2022;352:1048–1070. 36372385 10.1016/j.jconrel.2022.11.013

[13] Zindel J, Kubes P. DAMPs, PAMPs, and LAMPs in immunity and sterile inflammation. Annu Rev Pathol. 2020;15:493–518. 31675482 10.1146/annurev-pathmechdis-012419-032847

[14] Luster AD, Alon R, von Andrian UH. Immune cell migration in inflammation: Present and future therapeutic targets. Nat Immunol. 2005;6(12):1182–1190. 16369557 10.1038/ni1275

[15] Rajendran P, Chen YF, Chen YF, Chung LC, Tamilselvi S, Shen CY, Day CH, Chen RJ, Viswanadha VP, Kuo WW, et al. The multifaceted link between inflammation and human diseases. J Cell Physiol. 2018;233(9):6458–6471. 29323719 10.1002/jcp.26479 PMC13482531

[16] Esposito S, De Simone G, Boccia G, De Caro F, Pagliano P. Sepsis and septic shock: New definitions, new diagnostic and therapeutic approaches. J Glob Antimicrob Resist. 2017;10:204–212. 28743646 10.1016/j.jgar.2017.06.013

[17] Tang L, Zhang W, Liao Y, Wang W, Wu Y, Zou Z, Wang C. Decoding sepsis: Unraveling key signaling pathways for targeted therapies. Research. 2025;8:0811. 41041277 10.34133/research.0811 PMC12484860

[18] Patel P, Walborn A, Rondina M, Fareed J, Hoppensteadt D. Markers of inflammation and infection in sepsis and disseminated intravascular coagulation. Clin Appl Thromb Hemost. 2019;25:1076029619843338. 30991817 10.1177/1076029619843338 PMC6714897

[19] Bellaver B, Rocha AS, Souza DG, Leffa DT, De Bastiani MA, Schu G, Lukasewicz Ferreira PC, Venturin GT, Greggio S, Ribeiro CT, et al. Activated peripheral blood mononuclear cell mediators trigger astrocyte reactivity. Brain Behav Immun. 2019;80:879–888. 31176000 10.1016/j.bbi.2019.05.041

[20] Moraes CA, Zaverucha-do-Valle C, Fleurance R, Sharshar T, Bozza FA, d’Avila JC. Neuroinflammation in sepsis: Molecular pathways of microglia activation. Pharmaceuticals. 2021;14(5):416. 34062710 10.3390/ph14050416 PMC8147235

[21] Sonneville R, Verdonk F, Rauturier C, Klein IF, Wolff M, Annane D, Chretien F, Sharshar T. Understanding brain dysfunction in sepsis. Ann Intensive Care. 2013;3(1):15. 23718252 10.1186/2110-5820-3-15 PMC3673822

[22] Kuperberg SJ, Wadgaonkar R. Sepsis-associated encephalopathy: The blood-brain barrier and the sphingolipid rheostat. Front Immunol. 2017;8:597. 28670310 10.3389/fimmu.2017.00597 PMC5472697

[23] Peng X, Luo Z, He S, Zhang L, Li Y. Blood-brain barrier disruption by lipopolysaccharide and sepsis-associated encephalopathy. Front Cell Infect Microbiol. 2021;11: Article 768108. 34804998 10.3389/fcimb.2021.768108 PMC8599158

[24] Blom C, Deller BL, Fraser DD, Patterson EK, Martin CM, Young B, Liaw PC, Yazdan-Ashoori P, Ortiz A, Webb B, et al. Human severe sepsis cytokine mixture increases β2-integrin-dependent polymorphonuclear leukocyte adhesion to cerebral microvascular endothelial cells in vitro. Crit Care. 2015;19(1):149. 25882865 10.1186/s13054-015-0883-z PMC4409718

[25] Lamar CD, Hurley RA, Taber KH. Sepsis-associated encephalopathy: Review of the neuropsychiatric manifestations and cognitive outcome. J Neuropsychiatry Clin Neurosci. 2011;23(3):237–241. 21948885 10.1176/jnp.23.3.jnp237

[26] Ren C, Yao R-Q, Zhang H, Feng Y-W, Yao Y-M. Sepsis-associated encephalopathy: A vicious cycle of immunosuppression. J Neuroinflammation. 2020;17(1):14. 31924221 10.1186/s12974-020-1701-3 PMC6953314

[27] Hotchkiss RS, Karl IE. The pathophysiology and treatment of sepsis. N Engl J Med. 2003;348(2):138–150. 12519925 10.1056/NEJMra021333

[28] Hotchkiss RS, Monneret G, Payen D. Sepsis-induced immunosuppression: From cellular dysfunctions to immunotherapy. Nat Rev Immunol. 2013;13(12):862–874. 24232462 10.1038/nri3552 PMC4077177

[29] Hotchkiss RS, Opal S. Immunotherapy for Sepsis--a New Approach against an Ancient Foe. N Engl J Med. 2010;363(1):87–89. 20592301 10.1056/NEJMcibr1004371 PMC4136660

[30] Liu S, Li Y, She F, Zhao X, Yao Y. Predictive value of immune cell counts and neutrophil-to-lymphocyte ratio for 28-day mortality in patients with sepsis caused by intra-abdominal infection. Burns Trauma. 2021;9: Article tkaa 040. 10.1093/burnst/tkaa040 PMC7982795 33768121

[31] Wilson JK, Zhao Y, Singer M, Spencer J, Shankar-Hari M. Lymphocyte subset expression and serum concentrations of Pd-1/Pd-L1 in sepsis-pilot study. Crit Care. 2018;22(1):95. 29661225 10.1186/s13054-018-2020-2 PMC5902875

[32] Zhao P, Li J, He P, Wu Y, Zheng L, Yang X, Yang J, Fu Z, Xia Y, Chen N, et al. Nufip 1-mediated ribophagy alleviates panoptosis of Cd4^+^ T lymphocytes in sepsis via the CGAS-STING pathway. Research. 2026;9:0895. 10.34133/research.0895 PMC12454940 40995563

[33] Boomer JS, To K, Chang KC, Takasu O, Osborne DF, Walton AH, Bricker TL, Jarman SD II, Kreisel D, Krupnick AS, et al. Immunosuppression in patients who die of sepsis and multiple organ failure. JAMA. 2011;306(23):2594–2605. 22187279 10.1001/jama.2011.1829 PMC3361243

[34] Pan S, Lv Z, Wang R, Shu H, Yuan S, Yu Y, Shang Y. Sepsis-induced brain dysfunction: Pathogenesis, diagnosis, and treatment. Oxidative Med Cell Longev. 2022;2022:1328729. 10.1155/2022/1328729 PMC9433216 36062193

[35] Czempik PF, Pluta MP, Krzych ŁJ. Sepsis-associated brain dysfunction: A review of current literature. Int J Environ Res Public Health. 2020;17(16):5852. 32806705 10.3390/ijerph17165852 PMC7460246

[36] Fan J, Niu W, Guan R, Sun L, Wang M, Wang X, Zhang B, Hao X, Wu Q, Cheng Z, et al. Co-extracellular vesicles delivery system enhances immunochemotherapy for glioblastoma. Research. 2026;9:1219. 41924311 10.34133/research.1219 PMC13036365

[37] Vasiljevic B, Maglajlic-Djukic S, Gojnic M, Stankovic S, Ignjatovic S, Lutovac D. New insights into the pathogenesis of perinatal hypoxic-ischemic brain injury. Pediatr Int. 2011;53(4):454–462. 21077993 10.1111/j.1442-200X.2010.03290.x

[38] Kostandy BB. The role of glutamate in neuronal ischemic injury: The role of spark in fire. Neurol Sci. 2012;33(2):223–237. 22044990 10.1007/s10072-011-0828-5

[39] Luan YY, Yin CF, Qin QH, Dong N, Zhu XM, Sheng ZY, Zhang QH, Yao YM. Effect of regulatory T cells on promoting apoptosis of T lymphocyte and its regulatory mechanism in sepsis. J Interf Cytokine Res. 2015;35(12):969–980. 10.1089/jir.2014.0235 PMC4683547 26309018

[40] Yu P, Zhang X, Liu N, Tang L, Peng C, Chen X. Pyroptosis: Mechanisms and diseases. Signal Transduct Target Ther. 2021;6(1):128. 33776057 10.1038/s41392-021-00507-5 PMC8005494

[41] Shao BZ, Wang P, Bai Y. Editorial: Autophagy in inflammation related diseases. Front Pharmacol. 2022;13: Article 912487. 10.3389/fphar.2022.912487 PMC9117736 35600850

[42] Shadab M, Millar MW, Slavin SA, Leonard A, Fazal F, Rahman A. Autophagy protein Atg 7 is a critical regulator of endothelial cell inflammation and permeability. Sci Rep. 2020;10(1):13708. 32792588 10.1038/s41598-020-70126-7 PMC7426828

[43] Shen Y, Zhang Y, Du J, Jiang B, Shan T, Li H, Bao H, Si Y. CXCR5 down-regulation alleviates cognitive dysfunction in a mouse model of sepsis-associated encephalopathy: Potential role of microglial autophagy and the P38MAPK/NF-κB/STAT 3 signaling pathway. J Neuroinflammation. 2021;18(1):246. 34711216 10.1186/s12974-021-02300-1 PMC8554863

[44] Nakahira K, Haspel JA, Rathinam VA, Lee SJ, Dolinay T, Lam HC, Englert JA, Rabinovitch M, Cernadas M, Kim HP, et al. Autophagy proteins regulate innate immune responses by inhibiting the release of mitochondrial DNA mediated by the NALP3 Inflammasome. Nat Immunol. 2011;12(3):222–230. 21151103 10.1038/ni.1980 PMC3079381

[45] Samie M, Lim J, Verschueren E, Baughman JM, Peng I, Wong A, Kwon Y, Senbabaoglu Y, Hackney JA, Keir M, et al. Selective autophagy of the adaptor TRIF regulates innate inflammatory signaling. Nat Immunol. 2018;19(3):246–254. 29358708 10.1038/s41590-017-0042-6

[46] Symington JW, Wang C, Twentyman J, Owusu-Boaitey N, Schwendener R, Núñez G, Schilling JD, Mysorekar IU. ATG16L1 deficiency in macrophages drives clearance of uropathogenic *E. coli* in an IL-1β-dependent manner. Mucosal Immunol. 2015;8(6):1388–1399. 25669147 10.1038/mi.2015.7 PMC4532666

[47] Park SY, Shrestha S, Youn YJ, Kim JK, Kim SY, Kim HJ, Park SH, Ahn WG, Kim S, Lee MG, et al. Autophagy primes neutrophils for neutrophil extracellular trap formation during sepsis. Am J Respir Crit Care Med. 2017;196(5):577–589. 28358992 10.1164/rccm.201603-0596OC

[48] Maurer K, Reyes-Robles T, Alonzo F III, Durbin J, Torres VJ, Cadwell K. Autophagy mediates tolerance to *Staphylococcus aureus* alpha-toxin. Cell Host Microbe. 2015;17(4):429–440. 25816775 10.1016/j.chom.2015.03.001 PMC4392646

[49] Kashif AM, Ouyang Y, Li Y, Pan B. Netosis and pyroptosis of immune cells in sepsis. J Transl Int Med. 2025;13(4):318–327. 40861069 10.1515/jtim-2025-0035 PMC12371402

[50] Zhang X, Zhang Y, Yuan S, Zhang J. The potential immunological mechanisms of sepsis. Front Immunol. 2024;15:1434688. 39040114 10.3389/fimmu.2024.1434688 PMC11260823

[51] Singer M. The role of mitochondrial dysfunction in sepsis-induced multi-organ failure. Virulence. 2014;5(1):66–72. 24185508 10.4161/viru.26907 PMC3916385

[52] Vincent JL, Zhang H, Szabo C, Preiser JC. Effects of nitric oxide in septic shock. Am J Respir Crit Care Med. 2000;161(6):1781–1785. 10852744 10.1164/ajrccm.161.6.9812004

[53] Du Y, Zhu P, Li Y, Yu J, Xia T, Chang X, Zhu H, Li R, He Q. DNA-PKcs phosphorylates cofilin2 to induce endothelial dysfunction and microcirculatory disorder in endotoxemic cardiomyopathy. Research. 2024;7:0331. 38550779 10.34133/research.0331 PMC10976589

[54] Bone RC. The sepsis syndrome. Definition and general approach to management. Clin Chest Med. 1996;17(2):175–181. 8792059 10.1016/s0272-5231(05)70307-5

[55] Wang R, Han Q, Fan J, Xu Z, Liu W, Liu D, Li Y, Du J, Sun J, Zhang H, et al. Sepsis-induced endothelial barrier dysfunction: Mechanisms, pathology, and therapeutic advances. Research. 2025;8:0997. 41280722 10.34133/research.0997 PMC12635411

[56] Zhang L, Liu J, Mu S, Li H, Feng Y, Wang Y, Wang Y. Unfractionated heparin improves coagulation in sepsis by protecting glycocalyx of endothelia cells through inhibiting heparinase. J Transl Int Med. 2025;13(6):618–621. 41438473 10.1515/jtim-2025-0094 PMC12721356

[57] Buwalda M, Ince C. Opening the microcirculation: Can vasodilators be useful in sepsis? Intensive Care Med. 2002;28(9):1208–1217. 12209267 10.1007/s00134-002-1407-2

[58] Yu W, Zhao M, Guo X, Wang X, Wang J, Lyu Y, Shan A. Proteolytic-resistant self-assembling peptide nanofibers combat specific bacterial infections via trap and kill. Sci Adv. 2025;11(29):eadx0153. 40680130 10.1126/sciadv.adx0153 PMC12273793

[59] Beach MA, Nayanathara U, Gao Y, Zhang C, Xiong Y, Wang Y, Such GK. Polymeric nanoparticles for drug delivery. Chem Rev. 2024;124(9):5505–5616. 38626459 10.1021/acs.chemrev.3c00705 PMC11086401

[60] Zhang CY, Gao J, Wang Z. Bioresponsive nanoparticles targeted to infectious microenvironments for sepsis management. Adv Mater. 2018;30(43):e1803618. 30203430 10.1002/adma.201803618 PMC6197919

[61] Di J, Xie F, Xu Y. When liposomes met antibodies: Drug delivery and beyond. Adv Drug Deliv Rev. 2020;154–155:151–162. 10.1016/j.addr.2020.09.003 32926944

[62] Khatoon N, Zhang Z, Zhou C, Chu M. Macrophage membrane coated nanoparticles: A biomimetic approach for enhanced and targeted delivery. Biomater Sci. 2022;10(5):1193–1208. 35122479 10.1039/d1bm01664d

[63] Murao A, Brenner M, Aziz M, Wang P. Exosomes in sepsis. Front Immunol. 2020;11:2140. 33013905 10.3389/fimmu.2020.02140 PMC7509534

[64] Lee HP, Gaharwar AK. Light-responsive inorganic biomaterials for biomedical applications. Adv Sci. 2020;7(17):2000863. 10.1002/advs.202000863 PMC7507067 32995121

[65] Hussain S, Joo J, Kang J, Kim B, Braun GB, She Z-G, Kim D, Mann AP, Mölder T, Teesalu T, et al. Antibiotic-loaded nanoparticles targeted to the site of infection enhance antibacterial efficacy. Nat Biomed Eng. 2018;2(2):95–103. 29955439 10.1038/s41551-017-0187-5 PMC6015743

[66] Birk SE, Boisen A, Nielsen LH. Polymeric nano- and microparticulate drug delivery systems for treatment of biofilms. Adv Drug Deliv Rev. 2021;174:30–52. 33845040 10.1016/j.addr.2021.04.005

[67] Lelubre C, Vincent JL. Mechanisms and treatment of organ failure in sepsis. Nat Rev Nephrol. 2018;14(7):417–427. 29691495 10.1038/s41581-018-0005-7

[68] Klein DJ, Foster D, Walker PM, Bagshaw SM, Mekonnen H, Antonelli M. Polymyxin B hemoperfusion in endotoxemic septic shock patients without extreme endotoxemia: A post hoc analysis of the EUPHRATES trial. Intensive Care Med. 2018;44(12):2205–2212. 30470853 10.1007/s00134-018-5463-7 PMC6280819

[69] Ankawi G, Neri M, Zhang J, Breglia A, Ricci Z, Ronco C. Extracorporeal techniques for the treatment of critically ill patients with sepsis beyond conventional blood purification therapy: The promises and the pitfalls. Crit Care. 2018;22(1):262. 30360755 10.1186/s13054-018-2181-z PMC6202855

[70] London AS, Mackay K, Lihon M, He Y, Alabi BR. Gel filtration chromatography as a method for removing bacterial endotoxin from antibody preparations. Biotechnol Prog. 2014;30(6):1497–1501. 25079968 10.1002/btpr.1961

[71] Harm S, Lohner K, Fichtinger U, Schildböck C, Zottl J, Hartmann J. Blood compatibility—An important but often forgotten aspect of the characterization of antimicrobial peptides for clinical application. Int J Mol Sci. 2019;20(21):5426. 31683553 10.3390/ijms20215426 PMC6862080

[72] Harm S, Gabor F, Hartmann J. Low-dose polymyxin: An option for therapy of Gram-negative sepsis. Innate Immun. 2016;22(4):274–283. 26993088 10.1177/1753425916639120 PMC4834512

[73] Yuk SA, Kim H, Abutaleb NS, Dieterly AM, Taha MS, Tsifansky MD, Lyle LT, Seleem MN, Yeo Y. Nanocapsules modify membrane interaction of polymyxin B to enable safe systemic therapy of Gram-negative sepsis. Sci Adv. 2021;7(32):eabj 1577. 10.1126/sciadv.abj1577 PMC8346222 34362742

[74] Dwivedi DJ, Toltl LJ, Swystun LL, Pogue J, Liaw KL, Weitz JI, Cook DJ, Fox-Robichaud AE, Liaw PC. Prognostic utility and characterization of cell-free DNA in patients with severe sepsis. Crit Care. 2012;16(4):R151. 22889177 10.1186/cc11466 PMC3580740

[75] Li Z, Feng Y, Zhang S, Li T, Li H, Wang D, Hao K, He C, Tian H, Chen X. A multifunctional nanoparticle mitigating cytokine storm by scavenging multiple inflammatory mediators of sepsis. ACS Nano. 2023;17(9):8551–8563. 37129445 10.1021/acsnano.3c00906

[76] Gao N, Bai P, Fang C, Wu W, Bi C, Wang J, Shan A. Biomimetic peptide nanonets: Exploiting bacterial entrapment and macrophage rerousing for combatting infections. ACS Nano. 2024;18(37):25446–25464. 39240217 10.1021/acsnano.4c03669

[77] Yang H, Wang J, Wang X, Wang S, Xu J, Shan Q, Wang J, Ma X, Zhu Y. Nanofiber peptides for bacterial trapping: A novel approach to antibiotic alternatives in wound infections. Adv Healthc Mater. 2024;13(19):e2304657. 38607802 10.1002/adhm.202304657

[78] de Breij A, Riool M, Cordfunke RA, Malanovic N, de Boer L, Koning RI, Ravensbergen E, Franken M, van der Heijde T, Boekema BK, et al. The antimicrobial peptide SAAP-148 combats drug-resistant bacteria and biofilms. Sci Transl Med. 2018;10(423):eaan 4044. 10.1126/scitranslmed.aan4044 29321257

[79] Zou P, Huang L, Li Y, Liu D, Che J, Zhao T, Li H, Li J, Cui YN, Yang G, et al. Phase-separated nano-antibiotics enhanced survival in multidrug-resistant *Escherichia coli* sepsis by precise periplasmic EcDsbA targeting. Adv Mater. 2024;36(44):e2407152. 39279551 10.1002/adma.202407152

[80] Ha S, Kim J, Seo HW, Kim L, Yi YS, Seo SE, Kim KH, Kim S, An JE, Kim GJ, et al. Siderophore-functionalized nanodrug for treating antibiotic-resistant bacteria. ACS Nano. 2025;19(5):5131–5145. 39893588 10.1021/acsnano.4c06501

[81] McDonnell CJ, Garciarena CD, Watkin RL, McHale TM, McLoughlin A, Claes J, Verhamme P, Cummins PM, Kerrigan SW. Inhibition of major integrin α_V_β_3_ reduces *Staphylococcus aureus* attachment to sheared human endothelial cells. J Thromb Haemost. 2016;14(12):2536–2547. 27606892 10.1111/jth.13501

[82] McHale TM, Garciarena CD, Fagan RP, Smith SGJ, Martin-Loches I, Curley GF, Fitzpatrick F, Kerrigan SW. Inhibition of vascular endothelial cell leak following *Escherichia coli* attachment in an experimental model of sepsis. Crit Care Med. 2018;46(8):e805–e810. 29782355 10.1097/CCM.0000000000003219

[83] Nader D, Yousef F, Kavanagh N, Ryan BK, Kerrigan SW. Targeting internalized *Staphylococcus aureus* using vancomycin-loaded nanoparticles to treat recurrent bloodstream infections. Antibiotics. 2021;10(5):581. 34068975 10.3390/antibiotics10050581 PMC8156000

[84] Song C, Xu J, Gao C, Zhang W, Fang X, Shang Y. Nanomaterials targeting macrophages in sepsis: A promising approach for sepsis management. Front Immunol. 2022;13:1026173. 36569932 10.3389/fimmu.2022.1026173 PMC9780679

[85] Li Y, Zhang H, Chen C, Qiao K, Li Z, Han J, Han X, Li K, Lai K, Liu N, et al. Biomimetic immunosuppressive exosomes that inhibit cytokine storms contribute to the alleviation of sepsis. Adv Mater. 2022;34(19):e2108476. 35267211 10.1002/adma.202108476

[86] Ye M, Zhao Y, Wang Y, Xie R, Tong Y, Sauer JD, Gong S. Nad (H)-loaded nanoparticles for efficient sepsis therapy via modulating immune and vascular homeostasis. Nat Nanotechnol. 2022;17(8):880–890. 35668170 10.1038/s41565-022-01137-w PMC10044491

[87] Luk BT, Zhang L. Cell membrane-camouflaged nanoparticles for drug delivery. J Control Release. 2015;220(Pt B):600–607. 26210440 10.1016/j.jconrel.2015.07.019 PMC4688192

[88] Vijayan V, Uthaman S, Park I-K. Cell membrane-camouflaged nanoparticles: A promising biomimetic strategy for cancer theragnostics. Polymers. 2018;10(9):983. 30960908 10.3390/polym10090983 PMC6404000

[89] Tan S, Wu T, Zhang D, Zhang Z. Cell or cell membrane-based drug delivery systems. Theranostics. 2015;5(8):863–881. 26000058 10.7150/thno.11852 PMC4440443

[90] Rao L, Tian R, Chen X. Cell-membrane-mimicking nanodecoys against infectious diseases. ACS Nano. 2020;14(3):2569–2574. 32129977 10.1021/acsnano.0c01665 PMC7094139

[91] Parodi A, Quattrocchi N, van de Ven AL, Chiappini C, Evangelopoulos M, Martinez JO, Brown BS, Khaled SZ, Yazdi IK, Enzo MV, et al. Synthetic nanoparticles functionalized with biomimetic leukocyte membranes possess cell-like functions. Nat Nanotechnol. 2013;8(1):61–68. 23241654 10.1038/nnano.2012.212 PMC3751189

[92] Thamphiwatana S, Angsantikul P, Escajadillo T, Zhang Q, Olson J, Luk BT, Zhang S, Fang RH, Gao W, Nizet V, et al. Macrophage-like nanoparticles concurrently absorbing endotoxins and proinflammatory cytokines for sepsis management. Proc Natl Acad Sci USA. 2017;114(43):11488–11493. 29073076 10.1073/pnas.1714267114 PMC5664555

[93] Qu H, Wu J, Pan Y, Abdulla A, Duan Z, Cheng W, Wang N, Chen H, Wang C, Yang J, et al. Biomimetic nanomodulator regulates oxidative and inflammatory stresses to treat sepsis-associated encephalopathy. ACS Nano. 2024;18(41):28228–28245. 39367850 10.1021/acsnano.4c08157

[94] Khosrojerdi A, Soudi S, Hosseini AZ, Eshghi F, Shafiee A, Hashemi SM. Immunomodulatory and therapeutic effects of mesenchymal stem cells on organ dysfunction in sepsis. Shock. 2021;55(4):423–440. 32826813 10.1097/SHK.0000000000001644

[95] Lu L, Quan L, Li J, Yuan J, Nie X, Huang X, Dong H, Su Y, Huang Y, Kou Q, et al. Bioengineered stem cell membrane functionalized nanoparticles combine anti-inflammatory and antimicrobial properties for sepsis treatment. J Nanobiotechnol. 2023;21(1):170. 10.1186/s12951-023-01913-3 PMC10214628 37237294

[96] Vader P, Mol EA, Pasterkamp G, Schiffelers RM. Extracellular vesicles for drug delivery. Adv Drug Deliv Rev. 2016;106(Pt A):148–156. 26928656 10.1016/j.addr.2016.02.006

[97] Elsharkasy OM, Nordin JZ, Hagey DW, de Jong OG, Schiffelers RM, Andaloussi SE, Vader P. Extracellular vesicles as drug delivery systems: Why and how? Adv Drug Deliv Rev. 2020;159:332–343. 32305351 10.1016/j.addr.2020.04.004

[98] Zhou M, Li YJ, Tang YC, Hao XY, Xu WJ, Xiang DX, Wu JY. Apoptotic bodies for advanced drug delivery and therapy. J Control Release. 2022;351:394–406. 36167267 10.1016/j.jconrel.2022.09.045

[99] Hu M, Zhang J, Kong L, Yu Y, Hu Q, Yang T, Wang Y, Tu K, Qiao Q, Qin X, et al. Immunogenic hybrid nanovesicles of liposomes and tumor-derived nanovesicles for cancer immunochemotherapy. ACS Nano. 2021;15(2):3123–3138. 33470095 10.1021/acsnano.0c09681

[100] Lan H, Zhou Z, Hu Q, Xie Q, Li X, Tian T, Wang Y, Yang C, Kong L, Fu D, et al. Apoptotic body based biomimetic hybrid nanovesicles to attenuate cytokine storms for sepsis treatment. J Nanobiotechnol. 2024;22(1):775. 10.1186/s12951-024-03058-3 PMC11656764 39695736

[101] Li B, Niu Y, Ji W, Dong Y. Strategies for the CRISPR-based therapeutics. Trends Pharmacol Sci. 2020;41(1):55–65. 31862124 10.1016/j.tips.2019.11.006 PMC10082448

[102] van Haasteren J, Li J, Scheideler OJ, Murthy N, Schaffer DV. The delivery challenge: Fulfilling the promise of therapeutic genome editing. Nat Biotechnol. 2020;38(7):845–855. 32601435 10.1038/s41587-020-0565-5

[103] Osteikoetxea X, Silva A, Lázaro-Ibáñez E, Salmond N, Shatnyeva O, Stein J, Schick J, Wren S, Lindgren J, Firth M, et al. Engineered Cas9 extracellular vesicles as a novel gene editing tool. J Extracell Vesicles. 2022;11(5): Article e12225. 35585651 10.1002/jev2.12225 PMC9117459

[104] Konermann S, Lotfy P, Brideau NJ, Oki J, Shokhirev MN, Hsu PD. Transcriptome engineering with RNA-targeting type VI-D CRISPR effectors. Cell. 2018;173(3):665–676.e614. 29551272 10.1016/j.cell.2018.02.033 PMC5910255

[105] Li S, Li X, Xue W, Zhang L, Yang LZ, Cao SM, Lei YN, Liu CX, Guo SK, Shan L, et al. Screening for functional circular RNAs using the CRISPR-Cas13 system. Nat Methods. 2021;18(1):51–59. 33288960 10.1038/s41592-020-01011-4

[106] Li T, Zhang L, Lu T, Zhu T, Feng C, Gao N, Liu F, Yu J, Chen K, Zhong J, et al. Engineered extracellular vesicle-delivered CRISPR/CasRx as a novel RNA editing tool. Adv Sci. 2023;10(10):e2206517. 10.1002/advs.202206517 PMC10074121 36727818

[107] Kim YG, Choi B, Lee Y, Lee B, Kim H, Choi SH, Park OK, Kim Y, Baik S, Kim D, et al. Co-delivery of renal clearable cerium complex and synergistic antioxidant iron complex for treating sepsis. ACS Nano. 2024;18(43):29535–29549. 39419629 10.1021/acsnano.4c05902

[108] Li Y, Huang X, Qiao Q, Li Y, Han X, Chen C, Chen Y, Guo S, Zhang Y, Gao W, et al. Suppression of sepsis cytokine storm by *Escherichia coli* cell wall-derived carbon dots. Adv Mater. 2025;(12):37, Article e2414237. 10.1002/adma.202414237 39775885

[109] Zhu CL, Wang Y, Liu Q, Li HR, Yu CM, Li P, Deng XM, Wang JF. Dysregulation of neutrophil death in sepsis. Front Immunol. 2022;13: Article 963955. 36059483 10.3389/fimmu.2022.963955 PMC9434116

[110] Wang S, Lai X, Li C, Chen M, Hu M, Liu X, Song Y, Deng Y. Sialic acid-conjugate modified doxorubicin nanoplatform for treating neutrophil-related inflammation. J Control Release. 2021;337:612–627. 34332025 10.1016/j.jconrel.2021.07.044

[111] Zhang CY, Dong X, Gao J, Lin W, Liu Z, Wang Z. Nanoparticle-induced neutrophil apoptosis increases survival in sepsis and alleviates neurological damage in stroke. Sci Adv. 2019;5(11):eaax7964. 31723603 10.1126/sciadv.aax7964 PMC6834394

[112] Zhang T, Tian T, Lin Y. Functionalizing framework nucleic-acid-based nanostructures for biomedical application. Adv Mater. 2022;(46):34, Article e2107820. 10.1002/adma.202107820 34787933

[113] Chen Y, Chen X, Zhang B, Zhang Y, Li S, Liu Z, Gao Y, Zhao Y, Yan L, Li Y, et al. DNA framework signal amplification platform-based high-throughput systemic immune monitoring. Signal Transduct Target Ther. 2024;9(1):28. 38320992 10.1038/s41392-024-01736-0 PMC10847453

[114] Zhou M, Tang Y, Lu Y, Zhang T, Zhang S, Cai X, Lin Y. Framework nucleic acid-based and neutrophil-based nanoplatform loading baicalin with targeted drug delivery for anti-inflammation treatment. ACS Nano. 2025;19(3):3455–3469. 39817852 10.1021/acsnano.4c12917

[115] Essner R, Rhoades K, McBride WH, Morton DL, Economou JS. IL-4 down-regulates IL-1 and TNF gene expression in human monocytes. J Immunol. 1989;142(11):3857–3861. 2785566

[116] Woodward EA, Prêle CM, Nicholson SE, Kolesnik TB, Hart PH. The anti-inflammatory effects of interleukin-4 are not mediated by suppressor of cytokine signalling-1 (SOCS1). Immunology. 2010;131(1):118–127. 20406299 10.1111/j.1365-2567.2010.03281.x PMC2966764

[117] Schrijver DP, Röring RJ, Deckers J, de Dreu A, Toner YC, Prevot G, Priem B, Munitz J, Nugraha EG, van Elsas Y, et al. Resolving sepsis-induced Immunoparalysis via trained immunity by targeting interleukin-4 to myeloid cells. Nat Biomed Eng. 2023;7(9):1097–1112. 37291433 10.1038/s41551-023-01050-0 PMC10504080

[118] Zhou H, Coveney AP, Wu M, Huang J, Blankson S, Zhao H, O’Leary DP, Bai Z, Li Y, Redmond HP, et al. Activation of both TLR and NOD signaling confers host innate immunity-mediated protection against microbial infection. Front Immunol. 2018;9:3082. 30692992 10.3389/fimmu.2018.03082 PMC6339916

[119] Clarke TB, Davis KM, Lysenko ES, Zhou AY, Yu Y, Weiser JN. Recognition of peptidoglycan from the microbiota by NOD1 enhances systemic innate immunity. Nat Med. 2010;16(2):228–231. 20081863 10.1038/nm.2087 PMC4497535

[120] Zhao H, Lv X, Huang J, Huang S, Zhou H, Wang H, Xu Y, Wang J, Wang J, Liu Z. Two-phase releasing immune-stimulating composite orchestrates protection against microbial infections. Biomaterials. 2021;277: Article 121106. 34492581 10.1016/j.biomaterials.2021.121106

[121] Foster TJ. Immune evasion by staphylococci. Nat Rev Microbiol. 2005;3(12):948–958. 16322743 10.1038/nrmicro1289

[122] Lewis AJ, Richards AC, Mulvey MA. Invasion of host cells and tissues by uropathogenic bacteria. Microbiol Spectrum. 2016;4(6):10. 10.1128/microbiolspec.UTI-0026-2016 PMC5244466 28087946

[123] Döcke WD, Randow F, Syrbe U, Krausch D, Asadullah K, Reinke P, Volk HD, Kox W. Monocyte deactivation in septic patients: Restoration by IFN-gamma treatment. Nat Med. 1997;3(6):678–681. 9176497 10.1038/nm0697-678

[124] Netea MG, Joosten LA, Latz E, Mills KH, Natoli G, Stunnenberg HG, O’Neill LA, Xavier RJ. Trained immunity: A program of innate immune memory in health and disease. Science. 2016;352(6284):aaf1098. 27102489 10.1126/science.aaf1098 PMC5087274

[125] Hou X, Zhang X, Zhao W, Zeng C, Deng B, McComb DW, Du S, Zhang C, Li W, Dong Y. Vitamin lipid nanoparticles enable adoptive macrophage transfer for the treatment of multidrug-resistant bacterial sepsis. Nat Nanotechnol. 2020;15(1):41–46. 31907443 10.1038/s41565-019-0600-1 PMC7181370

[126] Tang C, Jing W, Han K, Yang Z, Zhang S, Liu M, Zhang J, Zhao X, Liu Y, Shi C, et al. mRNA-laden lipid-nanoparticle-enabled in situ CAR-macrophage engineering for the eradication of multidrug-resistant bacteria in a sepsis mouse model. ACS Nano. 2024;18(3):2261–2278. 38207332 10.1021/acsnano.3c10109

[127] Liu X, Xu W, Feng J, Wang Y, Li K, Chen Y, Wang W, Zhao W, Ge S, Li J. Adoptive cell transfer of piezo-activated macrophage rescues immunosuppressed rodents from life-threating bacterial infections. Nat Commun. 2025;16(1):1363. 39905015 10.1038/s41467-025-56460-2 PMC11794888

[128] Li Z, Luo B, Chen Y, Wang L, Liu Y, Jia J, Chen M, Yang S, Shi H, Dai L, et al. Nanomaterial-based encapsulation of biochemicals for targeted sepsis therapy. Mater Today Bio. 2025;33: Article 102054. 10.1016/j.mtbio.2025.102054 PMC12275963 40688672

[129] Thorley EV, Hatch J, Li M, Mashida SN, Castagnola E, Mesini A, Lehrnbecher T, Groll AH, Warris A, Ferreras-Antolin L. Liposomal amphotericin B prophylaxis in paediatrics: A systematic review. J Antimicrob Chemother. 2025;80(7):1792–1802. 40493030 10.1093/jac/dkaf171 PMC12209837

[130] Moghimi SM, Simberg D, Skotland T, Yaghmur A, Hunter AC. The interplay between blood proteins, complement, and macrophages on nanomedicine performance and responses. J Pharmacol Exp Ther. 2019;370(3):581–592. 30940695 10.1124/jpet.119.258012 PMC11047092

[131] Sellaturay P, Nasser S, Islam S, Gurugama P, Ewan PW. Polyethylene glycol (PEG) is a cause of anaphylaxis to the Pfizer/BioNTech mRNA COVID-19 vaccine. Clin Exp Allergy. 2021;51(6):861–863. 33825239 10.1111/cea.13874 PMC8251011

[132] Szebeni J. Complement activation-related pseudoallergy: A stress reaction in blood triggered by nanomedicines and biologicals. Mol Immunol. 2014;61(2):163–173. 25124145 10.1016/j.molimm.2014.06.038

[133] Zhang M, Xie J, Zhang P, Wang L, Wang Q, Yin W. Bidirectional regulatory roles in the immune modulation, organ dysfunction, and therapy of sepsis. Int J Nanomedicine. 2025;20:13527–13541. 41229676 10.2147/IJN.S542937 PMC12604511

[134] Hoffman A, Nizet V. The prospect of biomimetic immune cell membrane-coated nanomedicines for treatment of serious bacterial infections and sepsis. J Pharmacol Exp Ther. 2024;389(3):289–300. 38580449 10.1124/jpet.123.002095 PMC11125797

[135] Liu L, Li L, Wang T, Li Z, Yan B, Tan R, Zeng A, Ma W, Zhu X, Yin Z, et al. Recent nanoengineered therapeutic advancements in sepsis management. Front Bioeng Biotechnol. 2024;12:1495277. 39703795 10.3389/fbioe.2024.1495277 PMC11655211

[136] ISRCTN. Nanoselenium combined with glutamine versus glutamine alone for Sepsis. 2026. https://www.isrctn.com/ISRCTN88007295?q=&filters=recruitmentCountry:China&sort=date&offset=19&totalResults=843&page=1&pageSize=50

[137] Xiong MH, Bao Y, Yang XZ, Wang YC, Sun B, Wang J. Lipase-sensitive polymeric triple-layered nanogel for "on-demand" drug delivery. J Am Chem Soc. 2012;134(9):4355–4362. 22304702 10.1021/ja211279u

[138] Wang Y, Yuan Q, Feng W, Pu W, Ding J, Zhang H, Li X, Yang B, Dai Q, Cheng L, et al. Targeted delivery of antibiotics to the infected pulmonary tissues using ROS-responsive nanoparticles. J Nanobiotechnol. 2019;17(1):103. 10.1186/s12951-019-0537-4 PMC6777033 31581948

[139] Ni WW, Huang H, Yue QY, Yuan YD, Jiang SJ, Liu H, Zhang SM, Xiao WQ, Zhang M, Zhang YL, et al. Nanosensor-based pattern-generating probe accelerates sepsis diagnosis. ACS Nano. 2025;19(46):40061–40071. 41237320 10.1021/acsnano.5c14974

[140] Shuaibu II, Khan MA, Alkhamis D, Alkhamis A. In silico optimization of biomimetic nanoparticle kinetics for sepsis management: A computational pharmacology framework for rational design. medRxiv. 2026. 10.64898/2026.01.17.26344326

